# Effect of pancreas disease vaccines on infection levels and virus transmission in Atlantic salmon (*Salmo salar*) challenged with salmonid alphavirus, genotype 2

**DOI:** 10.3389/fimmu.2024.1342816

**Published:** 2024-03-07

**Authors:** Ragnar Thorarinsson, Anne Ramstad, Jeffrey C. Wolf, Hilde Sindre, Eystein Skjerve, Espen Rimstad, Øystein Evensen, Jose F. Rodriguez

**Affiliations:** ^1^ Elanco Animal Health, Bergen, Norway; ^2^ VESO Aqualab, Vikan, Namsos, Norway; ^3^ Experimental Pathology Laboratories Inc., Sterling, VA, United States; ^4^ Norwegian Veterinary Institute, Ås, Norway; ^5^ Faculty of Veterinary Medicine, Norwegian University of Life Sciences, Ås, Norway; ^6^ Elanco Canada Ltd., Mississauga, ON, Canada

**Keywords:** pancreas disease, salmonid alphavirus, Atlantic salmon, DNA vaccine, vaccine efficacy, viral shedding, disease transmission

## Abstract

Salmonid alphavirus (SAV) causes pancreas disease (PD), which negatively impacts farmed Atlantic salmon. In this study, fish were vaccinated with a DNA-PD vaccine (DNA-PD) and an oil-adjuvanted, inactivated whole virus PD vaccine (Oil-PD). Controls were two non-PD vaccinated groups. Fish were kept in one tank and challenged by cohabitation with SAV genotype 2 in seawater. Protection against infection and mortality was assessed for 84 days (Efficacy study). Nineteen days post challenge (dpc), subgroups of fish from all treatment groups were transferred to separate tanks and cohabited with naïve fish (Transmission study 1) or fish vaccinated with a homologous vaccine (Transmission study 2), to evaluate virus transmission for 26 days (47 dpc). Viremia, heart RT-qPCR and histopathological scoring of key organs affected by PD were used to measure infection levels. RT-droplet digital PCR quantified shedding of SAV into water for transmission studies. The Efficacy study showed that PD associated growth-loss was significantly lower and clearance of SAV2 RNA significantly higher in the PD-DNA group compared to the other groups. The PD-DNA group had milder lesions in the heart and muscle. Cumulative mortality post challenge was low and not different between groups, but the DNA-PD group had delayed time-to-death. In Transmission study 1, the lowest water levels of SAV RNA were measured in the tanks containing the DNA-PD group at 21 and 34 dpc. Despite this, and irrespective of the treatment group, SAV2 was effectively transmitted to the naïve fish during 26-day cohabitation. At 47 dpc, the SAV RNA concentrations in the water were lower in all tanks compared to 34 dpc. In Transmission study 2, none of the DNA-PD immunized cohabitants residing with DNA-PD-vaccinated, pre-challenged fish got infected. In contrast, Oil-PD immunized cohabitants residing with Oil-PD-vaccinated, pre-challenged fish, showed infection levels similar to the naïve cohabitants in Transmission study 1. The results demonstrate that the DNA-PD vaccine may curb the spread of SAV infection as the DNA-PD vaccinated, SAV2 exposed fish, did not spread the infection to cohabiting DNA-PD vaccinated fish. This signifies that herd immunity may be achieved by the DNA-PD vaccine, a valuable tool to control the PD epizootic in farmed Atlantic salmon.

## Graphical Abstract

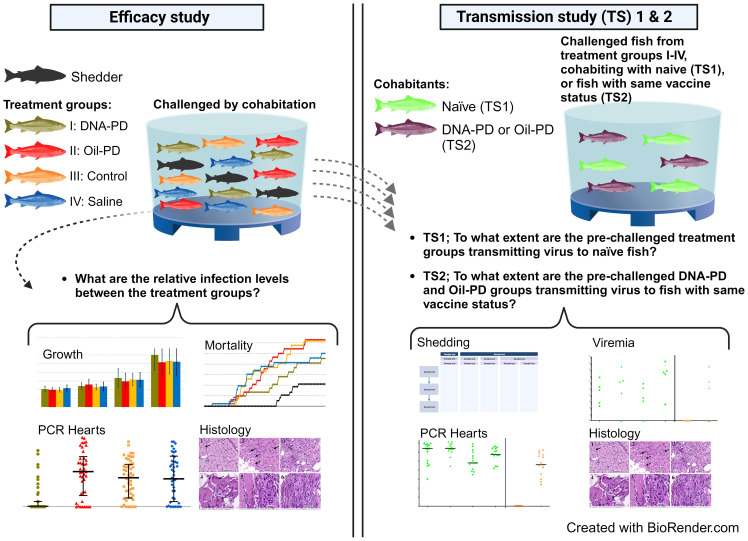

## 1 Introduction

Pancreas disease (PD) is an infectious and economically important disease affecting farmed Atlantic salmon (*Salmo salar*) and rainbow trout (*Oncorhynchus mykiss*) in seawater in Norway, Scotland and Ireland (1). The disease is caused by salmonid pancreas disease virus (SPDV) as designated by the International Committee on Taxonomy of Viruses (2), but today it is commonly called salmonid alphavirus (SAV) (3). Clinical manifestations of PD include increased mortality (4, 5), reduced growth rates (6, 7) and compromised filet quality at slaughter (8). Histological findings commonly include myocarditis and pancreatitis with loss of exocrine pancreatic tissue (pancreatic necrosis), as well as myositis in red and white skeletal muscle (9–12).

Seven different genotypes of SAV (SAV1-SAV7), have been described based on the nucleic acid sequences encoding the E2 glycoprotein and the non-structural protein nsP3 (13, 14). A cross-neutralization study revealed significant serological cross-reactivity among the SAV1 through SAV5 genotypes, while the findings for SAV6 were inconclusive (15). All the genotypes except SAV3 have been identified in Ireland and Scotland. SAV3 has only been confirmed in Norway (16) with an enzootic distribution confined to the southern west coast (SAV3 zone) (1, 17). Norway’s mid-region is a separate enzootic area with the PD cases mainly caused by the SAV2 (SAV2 zone) (1, 18). The geographic regions south of the SAV3 zone and north of the SAV2 zone are free of PD (19), and managed as surveillance zones in the national PD regulation (https://lovdata.no/dokument/SF/forskrift/2017-08-29-1318). This regulation aims to reduce the consequences of PD outbreaks within the enzootic zones, prevent the disease from establishing in the surveillance zones, and confine the spreading of the SAV genotypes. Transmission of SAV between neighboring seawater farms is believed to be horizontal through water currents (20–24), and with well-boat movements, including transfer of live fish (1). Current knowledge regarding the efficacy of fish vaccines curbing the spread and transmission of viral diseases in the aquatic environment is scarce (25). However, a pilot study evaluating the impact of a DNA vaccine against PD on the shedding and transmission of SAV revealed promising results (11). A reliable method to detect SAV RNA in seawater has recently been developed (26) and successfully employed in an SAV3 cohabitation challenge in seawater (27) as a valuable tool to better understand SAV infection and transmission dynamics.

In the present study, two commercially available PD vaccines and controls were administered to Atlantic salmon and later exposed to a SAV2 cohabitation challenge in seawater lasting 84 days (Efficacy study). At 19 days post challenge (dpc), a subset of fish from each treatment group were transferred to separate tanks and monitored for 28 days. These subsets were observed alone or with uninfected cohabitants added as either as naïve (Transmission Study 1, TS1) or immunized fish (Transmission Study 2, TS2). The key inquiries addressed in the study were; i) to compare the severity of the infection and protection against mortality for PD vaccinated versus control fish after challenge with SAV2; ii) to study whether vaccinated fish contribute to transmitting SAV2 to naïve cohabitant fish; and finally, iii) study if vaccinated fish contribute to transmission of SAV2 to PD immunized cohabitant fish.

## 2 Materials and methods

### 2.1 Fish and vaccination

**Figure 1:**
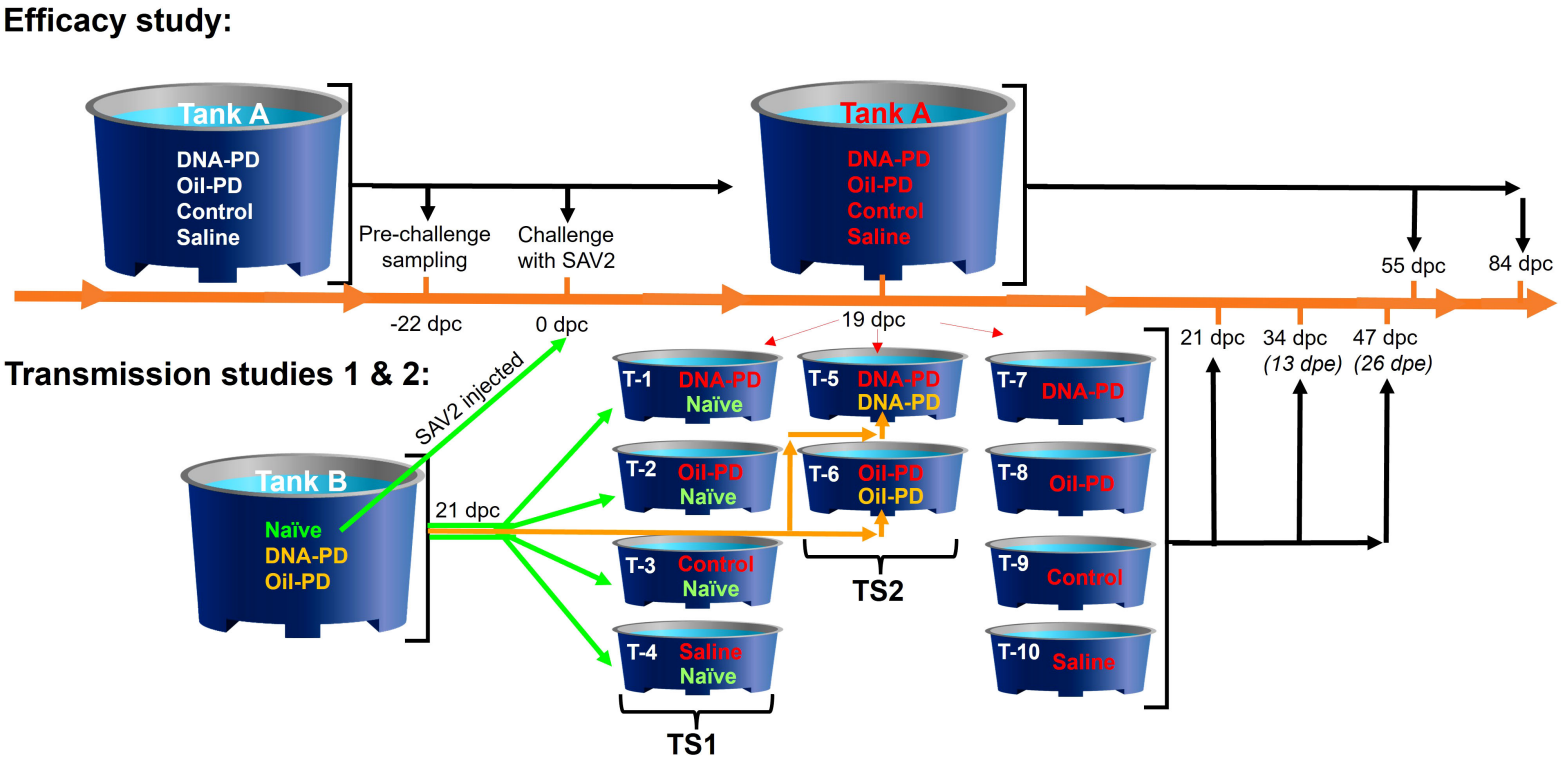
Design and timeline of the study in days post challenge (dpc) with A; the Efficacy study, and B; the transmission studies 1 (TS1) and 2 (TS2) above and below the timeline, respectively. The 4 groups (see **Table 1**) were PIT-tagged 2 weeks prior to vaccination and kept in Tank A. The pre-challenge sampling occurred 22 days before challenge (-22 dpc). The fish were challenged 9 days after transfer to seawater by adding naïve fish from Tank B that were injected i.p. with SAV2. Red font is used to indicate challenged fish groups. At 19, 55 and 84 dpc, fish were sampled from Tank A as detailed in **Table 2**. At 19 dpc, representative fish from each group (n=20) were transferred from Tank A to 10 tanks (T1-10) as shown in the figure. Two days later (=21 dpc) water samples for testing of viral quantification were taken from each of these tanks followed by addition healthy cohabitant fish from Tank B as follows; 20 naïve (green font) and 20 vaccinated fish (orange font) were added as cohabitants to each of Tanks 1-4 and Tanks 5-6, respectively. The sampling regime for the cohabitant fish in the transmission studies is depicted as days post exposure (dpe). The sampling regime for the transmission studies is detailed in **Table 3**.

The Atlantic salmon (Stofnfiskur Optimal strain) used in the study were reared from hatching at the VESO Aqualab’s hatchery (Fosslandsosen, Norway). Before the onset of the study, blood plasma was collected from 36 representative fish averaging ~20 grams, all of which tested negative for the presence of circulating antibodies using standard enzyme-linked immunosorbent assay (ELISA). The ELISA assays were based on methods described earlier for *Aeromonas salmonicida* subsp. *salmonicida* (28, 29) with exchange of the antigen coat on the ELISA plates to include *Vibrio salmonicida*, *Vibrio anguillarum* serotype O1 and 02, *V. ordalii*, *M. viscosa* and *Yersinia ruckeri*. Of the 36 fish analyzed by ELISA, 10 were further analyzed for viral RNA using validated and accredited (Norwegian Standard NS-EN ISO/IEC, 17025) RT-qPCR methodologies (PatoGen AS, Ålesund, Norway). Based on heart samples (see details in section 2.7), the fish were found negative for infectious salmon anaemia virus (ISAV), piscine myocarditis virus (PMCV), piscine reovirus (PRV), SAV and, based on kidney samples, these fish were also found negative for infectious pancreatic necrosis virus (IPNV). Healthy parr without visual deformities were size-graded and anaesthetized using tricaine mesylate at 200 mg/L for 40 seconds (Finquel vet., ScanVacc). The fish in tank A (**Figure 1**) were inserted intraperitoneally (i.p.) with a passive integrated transponder (PIT) tag and registered in VESO Aqualab’s database. Two weeks later, the fish were anaesthetized again and injected with vaccine combinations or sterile physiological saline (Saline) as listed in **Table 1**. The licensed vaccines used were administered in accordance with their respective summary of product characteristics (SPC). The weights of the PIT-tagged fish in tank A were registered and scanned during the vaccination process to register individual and treatment group identity. The fish in tank B were marked on a treatment group level only; as naïve fish by adipose fin-clipping (AFC), or by removal of the right- (RM) or left maxillary (LM) for their immunized counterparts as detailed in **Table 1**. The fish in the two tanks were transferred separately 13 weeks later to VESO-Aqualab’s experimental test facility (Namsos, Norway).

**Table 1:** Treatment, route of administration, dose and number of fish used in the Efficacy and the transmission studies (Tank A) and tank with supplementary fish (Tank B).

Group	Treatments	Route	Dose (mL)	No. of fish	
				Tank A	Tank B*
DNA-PD	Clynav (DNAV)^a^	i.m.	0.05	280	25**
	ALPHJA JECT micro 6^b^	i.p.	0.05		
	Alpha ERM Salar ^c^	i.p.	0.025		
Oil-PD	ALPHJA JECT micro 1 PD^d^ ALPHJA JECT micro 6^b^ Alpha ERM Salar ^c^	i.p. i.p. i.p.	0.05 0.05 0.025	277	25***
Control	ALPHJA JECT micro 6^b^ Alpha ERM Salar ^c^	i.p. i.p.	0.05 0.025	256	n.a.
Saline	Physiological saline	i.p.	0.05	260	n.a.
Naïve	No treatment	n.a.	n.a.	n.a.	310****

^a^Produced by Elanco Animal Health. Clynav contains pUK-SPDV-poly2#1 DNA plasmid coding for SPDV proteins. See SPC (https://www.ema.europa.eu/en/documents/product-information/clynav-epar-product-information_en.pdf).

^b^Produced by Pharmaq. ALPHJA JECT micro 6 (AJm6) is a hexavalent OAV containing inactivated Aeromonas salmonicida subsp. salmonicida, Aliivibrio salmonicida, Listonella anguillarum serotype O1, L. anguillarum serotype O2a, Moritella viscosa and infectious pancreatic necrosis virus (IPNV). See SPC (https://vmd.defra.gov.uk/productinformationdatabase/files/SPC_Documents/SPC_1566772.PDF)

^c^Produced by Pharmaq. Alpha ERM Salar is a water-based monovalent bacterin containing inactivated Yersinia ruckeri, serotype O1b. See SPC only available in Norwegian (https://www.legemiddelsok.no/_layouts/15/Preparatomtaler/Spc/19-12915.pdf).

^d^Produced by Pharmaq. “AJm 1 PD” = ALPHA JECT micro 1-PD is an OAV containing formaldehyde inactivated culture of SPDV. See SPC (https://vmd.defra.gov.uk/productinformationdatabase/files/SPC_Documents/SPC_916517.PDF).

“i.m.” = intramuscular, “i.p.” = intraperitoneal, “n.a.” = not applicable.

*Fish in Tank B were marked for identification by; ** cutting right maxillary (RM), *** cutting the left maxillary (LM) or **** adipose fin clipping (AFC) used as injected shedders, as naïve cohabitant fish in TS1, as vaccinated cohabitant fish in TS2 and to serve as negative controls in the histological analysis.

### 2.2 Husbandry, feeding and smoltification

The fish were reared at 12 ± 1°C in the hatchery in two 1.5-meter diameter tanks. At the experimental facility, the fish were maintained at 13 ± 1°C in either a 2-meter (tank A) or a 1.5-meter (tank B) tank with tube overflow system (**Figure 1**). Flow rates were adjusted to ensure that oxygen saturation levels near the outlet remained ≥70%. Removing dead fish and cleaning the tanks was done daily. The fish were starved for minimum 24 hours prior to handling or sampling. All fish were kept sedated to minimize stress during sampling using AQUI-S VET (isoeugenol, MSD Animal Health) according to the product’s label specifications. Moribund and sampled fish removed from the tanks were euthanized using an overdose of benzocaine chloride. By use of automated feeders, the fish were fed a standard commercial extruded pellets without any immunostimulant supplements (Skretting) throughout the study. Feeding rates were 2% body weight per day prior to challenge in Tank A and for the fish in the 10 tanks used in the transmission study. The fish in Tank A were fed *ad libitum* during the 84-day challenge period. Standard photomanipulation was applied using 12-hours light and 12-hours darkness (12:12) exposure for 6 weeks followed by continuous 24-hour light exposure (24:0) for another 6 weeks before seawater exposure (salinity maintained at 32 ± 3‰). The photomanipulation process was timed so that the fish in tank A were ready for exposure to seawater 1-2 weeks earlier than their counterparts in tank B. All fish were handled in accordance with Norwegian “Regulation on Animal Experimentation”, and the study protocol was approved before start-up by the Norwegian Animal Research Authority (FOTS ID, 27332).

### 2.3 Efficacy study

Fish in Tank A were sampled 22 days before the challenge, still in freshwater, 1030 degree-days (dd) post vaccination (**Table 2**). The challenge was carried out, 1476 dd post vaccination, 9 days after seawater exposure. Briefly, a total of 200 naïve AFC smolts residing in freshwater in tank B were anaesthetized and injected i.p. with 0.1 ml of SAV2 inoculum derived from a PD outbreak in Romsdal, 2011 (Isolate 1; Taksdal et al. (7), GenBank ref. HE863664, MZ395641) containing 3.52 × 10^4^ TCID_50_/ml, and dropped into tank A containing seawater. These fish (shedders) represented ~19% of the total number of fish in Tank A at the onset of the challenge period (0 days post challenge (dpc)). Dead and moribund fish were removed daily, and their PIT-tag identity scanned and registered in the database throughout the study. Heart samples were collected from all dead PIT-tagged fish between 27 and 53 dpc, and analyzed for relative quantities of SAV RNA by RT-qPCR. Head-kidney smears from the dead PIT-tagged fish were cultured on blood agar with 2% NaCl and incubated at 22°C between 48 and 96 hours. Culture growth was evaluated to detect possible bacterial causes of mortality. Samples were collected at 19, 55 and 84 dpc as shown in **Figure 1**; **Table 2**. The cumulative percent mortality during the challenge period was calculated with the denominator adjusted based on the number of fish removed due to samplings at 19 and 55 dpc (**Table 2**) and by transfer to the 10 tanks used for the transmission studies at 19 dpc (**Figure 1**).

**Table 2:** Sampling regime for the Efficacy study (Tank A) for each of the 4 groups (DNA-PD, Oil-PD, Control and Saline).

Sampling objective	Number of fish per group			
	Before challenge^a,b^	19 dpc	55 dpc	84 dpc^b^
Neutralization test	30	–	–	42-49
Side effects i.p.	50	–	–	–
Viremia	–	20	20	42-49
PCR	–	20	20	42-49
Histology^c^	–	20	20	42-49
Measure length and weight	75	20	20	102-104

^a^ Sampled in freshwater 22 days before challenge (-22 dpc).

^b^ The fish collected from each group were used for the different analyses.

^c^ Includes evaluations of heart, pancreas, red and white skeletal muscle.

### 2.4 Transmission studies

The experimental design and sampling schedule employed in the transmission studies are illustrated in **Figure 1**. At 19 dpc, fish from each treatment group, hereinafter referred to as the ‘pre-challenged fish’, were transferred from tank A into 10 tanks of 1-meter diameter with equal flow rates. At 21 dpc, non-infected fish from tank B, either as naïve (TS1) or vaccinated fish (TS2), were transferred to six of the shedder tanks as shown in **Figure 1**. These fish are hereinafter referred to as ‘naïve’ or the ‘vaccinated cohabitants’. With 40 fish in six shedder tanks (1-6) and 20 fish in the remaining four tanks (7-10) at 21 dpc, flow rates were biomass adjusted in the 10 tanks to 1.56 liters/kg fish/minute. As this flow rate resulted in oxygen saturation levels near the tanks outlets of 62%, the flow rate was increased at 23 dpc to 1.86 liters/kg fish/minute in the 10 tanks and until the end of the transmission studies at 47 dpc. The sampling regime of the transmission studies is detailed in **Table 3**.

**Table 3:** Sampling regime for the transmission studies in Tanks 1-10 (see **Figure 1**) in days post challenge (dpc).

Sampling objective	Tanks 1 -10			
	21 dpc	13 dpe	26 dpe	47 dpc
SAV in water	One sample/tank	One sample/tank	One sample/tank	
Viremia	n.a.	5^a^	15^a^	19−20^b^
PCR	n.a.	5^a^	15^a^	19−20^b^
Histology^c^	n.a.	5^a^	15^a^	19−20^b^

Days post challenge (dpc) denote the pre-challenged fish and days post exposure (dpe) for the cohabitant fish added to tanks 1-6 at 21 dpc.

^a^Includes cohabitants added as naïve (TS1) (tanks 1-4) or immunized (TS2) fish (tanks 5 and 6) at 21 dpc.

^b^Includes pre-challenged fish. “n.a”, not applicable.

^c^Includes evaluations of heart and pancreas only.

### 2.5 Neutralization test

For the neutralization test, blood was collected from the caudal vein of euthanized fish using heparin-coated vacutainers and placed into crushed ice immediately thereafter (**Table 2**). After centrifugation at, 1000 x g for 10 minutes, plasma samples were retrieved and stored at -80°C until use. The neutralization test was performed as previously described (30) with some modifications. CHSE-214 cells, derived from Chinook salmon (Oncorhynchus tshawytscha) embryo (ECACC CB_91041114), were grown as recommended in section 4.3 of the WOAH manual (https://www.woah.org/fileadmin/Home/eng/Health_standards/aahm/current/2.3.08_SAV.pdf). Fully confluent cells were trypsinized, seeded in a 96-well cell plate, and grown until 80% confluency. Starting with 1:20 dilution, two-fold dilution series of plasma were incubated with SAV2 (same isolate as used in the challenge – see section 2.3) for two hours and then seeded in two parallel wells in the prepared CHSE-214 96 well plate. After 3-4 days of incubation at 15°C, the cell layer was fixed with 80% acetone. SAV-infected cells were visualized using an indirect immunofluorescence test according to the procedure described by Falk et al. (31), but with the use of monoclonal antibody 17H23 directed against the E2 glycoprotein of SAV (32) as the primary antibody and with biotin labelled goat anti-mouse Ig (RayBiotech) and FITC-labelled streptavidin (Invitrogen) as the secondary amplification step. The number of positive cells were counted using a fluorescence microscope. Neutralizing activity was defined as present when more than a 50% reduction in infected cells relative to control wells was observed, as previously described (33). Neutralizing activity in plasma diluted ≥1:20 was recorded as a positive result.

### 2.6 Vaccine side effects

Intraperitoneal side-effects were evaluated in a blinded manner and the degree of visceral adhesions scored on a progressive scale from 0 (no adhesions) to 6 as previously described (34). The number of fish per group used for the side effect evaluations is shown in **Table 2**.

### 2.7 SAV RNA in water and hearts

In the transmission studies, water samples were collected at 3 time points (**Table 3**) 5 minutes after the tank’s inlet flow had been turned off. The water was collected using, 1000 ml polyethylene terephthalate bottles (VWR International, LLC), and shipped refrigerated overnight for next-day processing. The relative amount of SAV2 RNA in water samples was quantified as previously described using an RT-ddPCR (RT-droplet digital PCR) method (26). In short, 1000 ml of water from each tank and timepoint was filtrated through a negatively charged nitrocellulose MF hydrophilic membrane filter (MF-Millipore^®^) using a peristaltic pump at a flow rate of 200 ml/min. The filter was placed in 2,4 ml lysis buffer in a 60 mm petri-dish and put on a shaker for 30 min at 600 rpm. The membrane filter was removed, and the buffer-sample frozen at -80°C until further analysis. To purify nucleic acids, SAV RNA was extracted using a fully automated MagNA Pure 96 Instrument. The extracted RNA was analyzed by RT-ddPCR to quantify the amount of SAV RNA present.

The RT-qPCR (PCR) analysis measured the relative amounts of SAV RNA in the hearts of fish (**Tables 2**, **3**). In short, a small tangential portion of the heart (~2 × 2 mm) was cut along the sagittal plane and placed into a tube containing RNAlater (Thermo Fischer Scientific, Waltham, MA, USA). Samples in RNAlater were stored overnight at 4°C and then frozen at -80°C until use. The PCR analysis was carried out using a validated and ISO17025-accredited method (Patogen AS, Ålesund, Norway) previously described (35). The cut-off Cq-value was set to 37.

### 2.8 Viremia

The TCID_50_ of SAV in plasma samples was determined as previously described (36) with some modifications. In brief, ten-fold dilution series of plasma were seeded on CHSE-214 cells in four parallel wells (96 well plates). The cell layer was fixed with 80% acetone after 7 days of incubation at 15°C. SAV-infected cells were visualized using an indirect immunofluorescence assay as described above. The TCID_50_ titres were calculated as described by Kärber (37).

### 2.9 Histopathology

Formalin-fixed samples of heart, pancreas, red and white skeletal muscle were processed for paraffin embedding. For each fish, a single sagittal section was obtained through the heart, which included the ventricle, atrium and bulbus arteriosus. To evaluate the pancreas, a single transverse section was acquired through the pyloric ceca; each of these sections invariably contained multiple islands of exocrine and endocrine pancreatic tissue. Skeletal muscle samples from the lateral line region were microtomed to provide one transverse section and two longitudinal sections per fish, each containing red and white skeletal muscle. Histologic sections (4-6 µm thick) were mounted on glass slides and stained with hematoxylin and eosin using standard methodology. All slides were examined in a blinded manner using brightfield microscopy at various magnifications (20x – 400x) by an experienced anatomic pathologist, certified by the American College of Veterinary Pathologists. Histopathological changes associated with SAV infection were recorded as previously reported (11) and detailed in **Table 4**. Each characteristic was scored for severity using a 0-3 scale: 0 = not remarkable, 1 = mild changes, 2 = moderate changes, and 3 = severe changes. Representative pictures and descriptions of the histopathological changes (Grades) for heart, pancreas and muscle used in this study are included as **Supplementary Figures S1**–**S3**.

**Table 4:** The semi-quantitative scoring system applied for histopathological evaluation post SAV2 exposure.

Organ	Score	Necrosis	Inflammation	Fibrosis	Muscle Regeneration	Tissue Loss
**Heart**	1	1 necrotic myocyte per section to 1 necrotic myocyte per 40x field	1-4 discontinuous layers of epicardial leukocytic infiltrates	Collagen fibers < 10% of muscle tissue	Regeneration < 10% of muscle tissue	n.a.
	2	2 to 4 necrotic myocytes per 40x field	5-10 layers of epicardial leukocytic infiltrates, +/- myocardial infiltrates	Collagen fibers ≥ 10% but ≤ 50% of muscle tissue	Regeneration ≥ 10% but ≤ 50% of muscle tissue	n.a.
	3	> 4 necrotic myocytes per 40x field	> 10 layers of epicardial leukocytic infiltrates, +/- myocardial infiltrates	Collagen fibers > 50% of muscle tissue	Regeneration > 50% of muscle tissue	n.a.
**Skeletal Muscle** **(red and white scored individually)**	1	1 necrotic myocyte per section to 1 necrotic myocyte per 20x field	Leukocytic infiltrates < 10% of muscle tissue	Collagen fibers < 10% of muscle tissue	Regeneration < 10% of muscle tissue	n.a.
	2	2 to 4 necrotic myocytes per 20x field	Leukocytic infiltrates ≥ 10% but ≤ 50% of muscle tissue	Collagen fibers ≥ 10% but ≤ 50% of muscle tissue	Regeneration ≥ 10% but ≤ 50% of muscle tissue	n.a.
	3	> 4 necrotic myocytes per 20x field	Leukocytic infiltrates > 50% of muscle tissue	Collagen fibers > 50% of muscle tissue	Regeneration > 50% of muscle tissue	n.a.
**Exocrine Pancreas**	1	< 10% of acinar tissue necrotic	Leukocytic infiltrates < 10% of pancreatic tissue	Collagen fibers < 10% of acinar tissue	n.a.	< 50% of acinar tissue lost
	2	≥ 10% to ≤ 50% of acinar tissue necrotic	Leukocytic infiltrates ≥ 10% but ≤ 50% of pancreatic tissue	Collagen fibers ≥ 10% but ≤ 50% of acinar tissue	n.a.	≥ 50% of acinar tissue lost, but some acinar tissue remains
	3	> 50% of acinar tissue necrotic	Leukocytic infiltrates > 50% of pancreatic tissue	Collagen fibers > 50% of acinar tissue	n.a.	All acinar tissue lost

“n.a.”, not applicable.

### 2.10 Statistical analysis

Initial analysis was carried out using Pivot tabulations and graphs in Excel^®^. The data were transferred and analyzed using Stata/MP 17 for Windows (StataCorp, College Station, TX). The weight gain prior to the challenge was analyzed using ANOVA and a linear regression platform with the treatment group and start weight as explanatory variables. Weight gain post challenge was analyzed using linear regression. Start length was also included as a predictor but dropped as no influence was found. The intraperitoneal side effects levels of the immunized were analyzed using Fisher’s exact test. Only the DNA-PD group produced neutralization titers against SAV2 prior to the challenge while all the groups had high and similar titers at the end of the challenge period (84 dpc). Statistical analysis of this data was therefore deemed unnecessary.

Median regression analysis was used to compare SAV viremia levels and RT-qPCR Cq-values of the hearts. The concentrations of SAV RNA in water between the groups at the first timepoint (21 dpc) were also compared using median regression analysis. The cohabitant fish thereafter added to tanks 1-6 rendered the subsequent SAV RNA in water data limited to descriptive interpretation.

The histopathology data were first examined using tabular and graphical techniques followed by ordinal logistic regression analysis. Two analyses were undertaken by first splitting data across cohabitant (naïve and vaccinated) and pre-challenged fish estimating only vaccine effects. As the different cohabitant combinations influence the infectious dynamics, a second analysis was carried out splitting the data across cohabitant patterns (naïve + pre-challenged, vaccinated + pre-challenged and pre-challenged only) and estimating the effects of vaccine and cohabitants. These analyses are complementary. Linear and median regression models are presented using coefficients with 95% Confidence intervals (95% CI) and corresponding p-values. Ordinal regression models are presented with Odds ratios with 95% CI and corresponding p-values. The term significant in the text refers to a p-value <0.05.

## 3 Results

### 3.1 Pre-challenge

**Figure 2:**
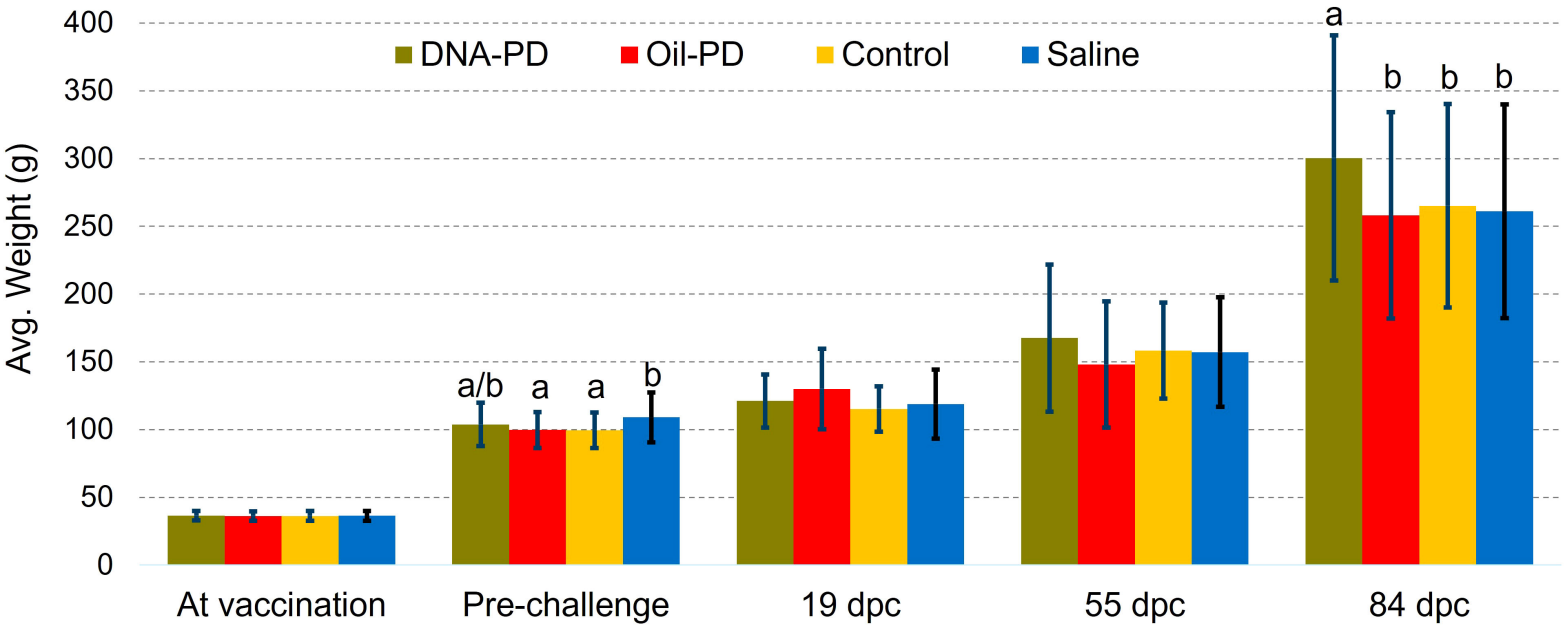
Average weights ± one standard deviation at vaccination (n=256-280), pre-challenge (n =75), 19 (n=20), 55 (n=20) and 84 dpc (n=102-104). Different letters (a, b) denote significant differences (Linear regression analysis p<0.015).

The mean weight of all the fish in tank A (n=1073) at vaccination was 36.4g ±3.6g (min/max 28.0/46.4). When weighed 22 days before the challenge (after, 1030 dd), the Saline group had gained more weight than the immunized groups (**Figure 2**), as expected when oil-adjuvanted vaccines (OAVs) are used. No significant difference in weight gain (n=75) or visceral adhesions scores (n=50) was found at this time between the immunized groups with mean adhesions scores of 1.56, 1.60 and 1.44 for the DNA-PD, Oil-PD and Control groups, respectively. These side effect levels are within the range reported in the SPC of the OAVs used. Before the challenge, plasma neutralization titers of SAV2 were only observed for the DNA-PD group, (n=30) with 83% prevalence and titers ranging from 1:20 to 1:640 (**Supplementary Figure S4**). A total of 17 fish (1.6%) died during the immunization period until the challenge, but there were no indications of infectious disease.

### 3.2 Efficacy study

#### 3.2.1 Severity of infection for PD vaccinated and control fish after challenge with SAV2

**Figure 3:**
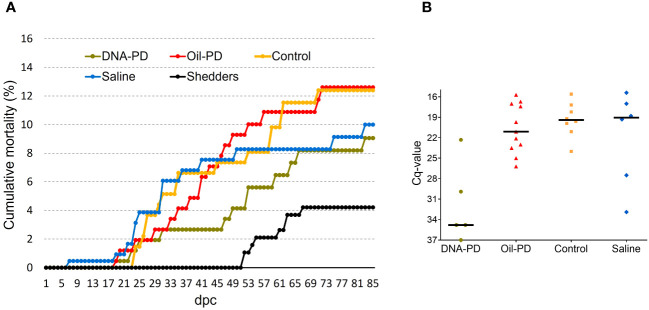
**(A)** Cumulative percent mortality of the treatment groups post challenge **(B)** Scattered dot plot showing Cq-values of the hearts of fish that died between 27 and 53 dpc.

Post challenge, there were no significant weight differences between the groups at 19 and 55 dpc, but at 84 dpc the DNA-PD group had significantly greater weight (p=0.001) than the other groups (300g versus ≤265g) (**Figure 2**). The cumulative mortality post challenge ranged from 9.1% in the DNA-PD group to 12.6% in the Oil-PD group. As shown in the graph, the fish in the DNA-PD group died later than those in the other groups, but no statistical differences in total mortality were found (**Figure 3A**). The qPCR results of the hearts of fish that died between 27 and 53 dpc revealed low Cq-values except for the DNA-PD group (**Figure 3B**).

As previously reported from a SAV3 challenge experiment (38) and field outbreaks (39, 40), consistently strong neutralizing capacity was measured in the plasmas of fish in all treatment groups at the end of the challenge period, 84 dpc, reaching titers of 1:2560 (**Supplementary Figure S4**).

**Figure 4:**
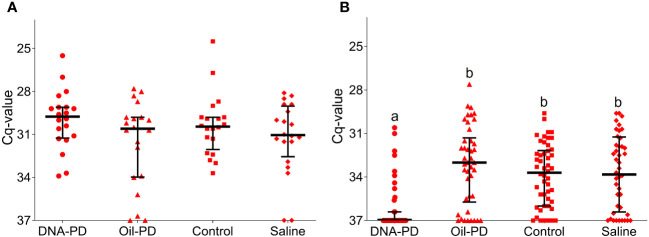
RT-qPCR SAV2 of the hearts showing individual Cq-values from the Efficacy study including medians (horizontal lines) and interquartile ranges. **(A)** fish sampled 55 dpc (n=20). **(B)** fish sampled 84 dpc (n=42-50). Different letters (a, b) denote significant differences between the DNA-PD and the other groups (Median regression analysis p<0.001). The vertical axis value of 37 denotes the cut-off cycle for the PCR.

Data gathered from fish sampled at 19 dpc, including viremia, PCR and histopathology results revealed a low prevalence of SAV infection and early stage of PD. At this timepoint, 13 fish (16%) were viremic with 0, 2, 5 and 6 fish from the DNA-PD, Oil-PD, Control and Saline groups, respectively. The viremia levels ranged from ~100 to 3.3 × 10^5^ TCID_50_/ml of plasma (**Supplementary Figure S5**). Only two fish with viremia were found in the samples collected at 55 dpc and both were from the DNA-PD group (3.2 × 10^5^ and 1.3 × 10^7^ TCID_50_/ml). The fact that none of the fish sampled at 84 dpc had viremia suggests that the viremic phase peaked between 19 and 55 dpc. Only 3 of the 80 fish sampled at 19 dpc had positive PCR results from heart samples (one from Oil-PD and two from Control; these fish were also viremic), all with Cq-values >29 (data not shown). At 55 dpc, 76 of the 80 fish sampled were PCR-positive in the heart with relatively high and similar Cq-values across the groups with medians between 30 and 31 (**Figure 4A**). The Cq-values increased further in the hearts at 84 dpc. At this timepoint, the DNA-PD group had significantly (p<0.001) lower levels of SAV RNA, and only 30% were PCR-positive, while for the other groups between 82 to 88% were PCR-positive (**Figure 4B**).

**Figure 5:**
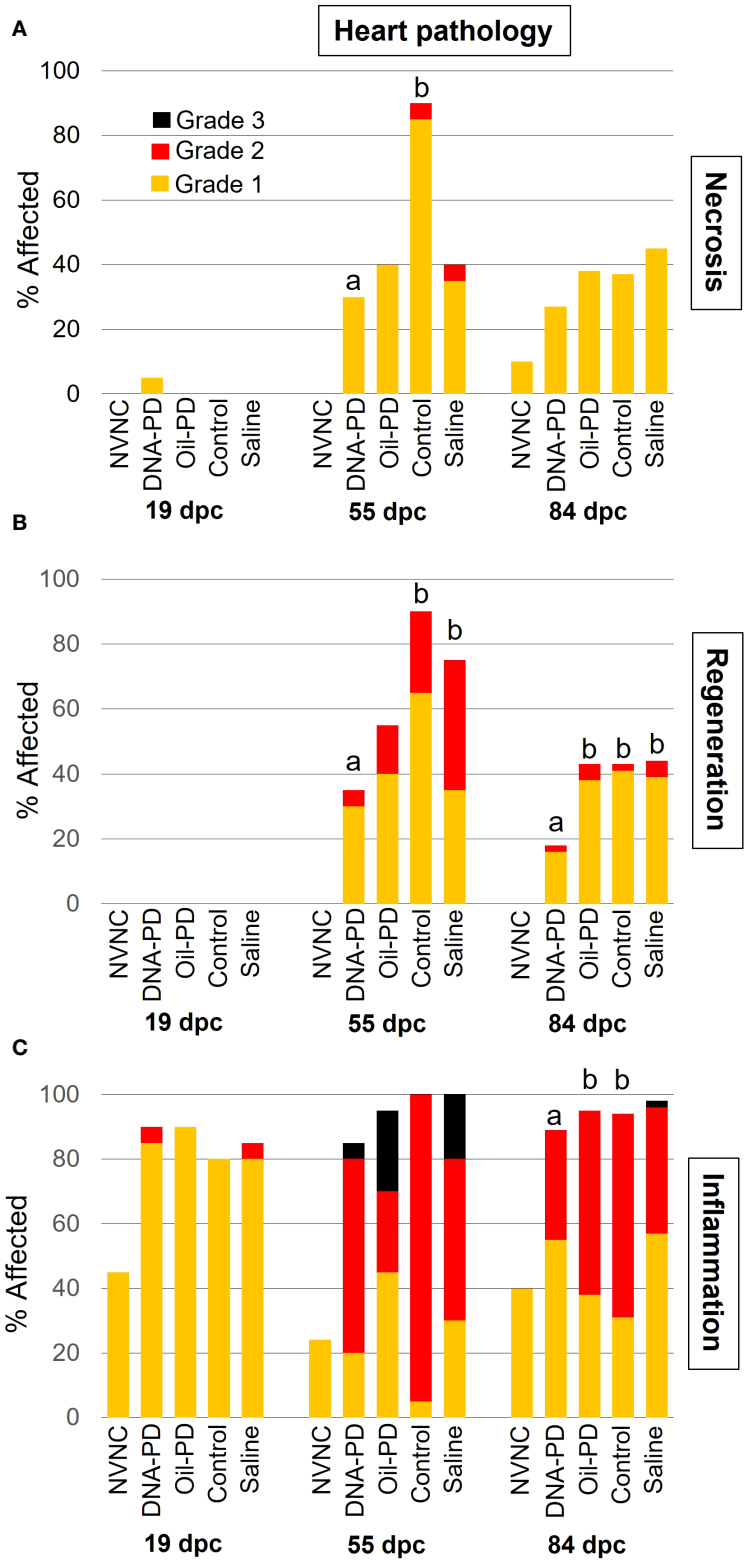
The prevalence and severity of necrosis **(A)**, cardiac myocyte regeneration **(B)**, and inflammation **(C)** in hearts sampled at 19 (n= 20), 55 (n=20) and 84 (n= 42-49) dpc. NVNC indicates non-vaccinated and non-challenged controls (n=10). Different letters (a, b) denote statistically significant differences between the DNA-PD and the other groups within each pathology criterium and each sampling point (Ordinal logistic regression p<0.05) with NVNC excluded. Note that the total height of each bar represents the overall prevalence of each finding.

At 19 dpc, no cardiac necrosis or regeneration was observed whereas In contrast, a high prevalence (≥80%) of predominantly grade 1 inflammation was found in all the groups, and markedly higher than found in the non-vaccinated and non-challenged control (NVNC) counterparts (**Figure 5**). The prevalence and severity of cardiac necrosis, inflammation, and regeneration peaked at 55 dpc, then tapered off slightly by 84 dpc (**Figure 5**). Levels of cardiac regeneration were significantly lower in the DNA-PD group compared to the Control and Saline groups at 55 dpc and the other three groups at 84 dpc (**Figure 5B**). No differences in cardiac inflammation were found between the groups at 55 dpc. In comparison, at 84 dpc, the DNA-PD group exhibited significantly milder inflammation than the Oil-PD and Control groups (**Figure 5C**).

**Figure 6:**
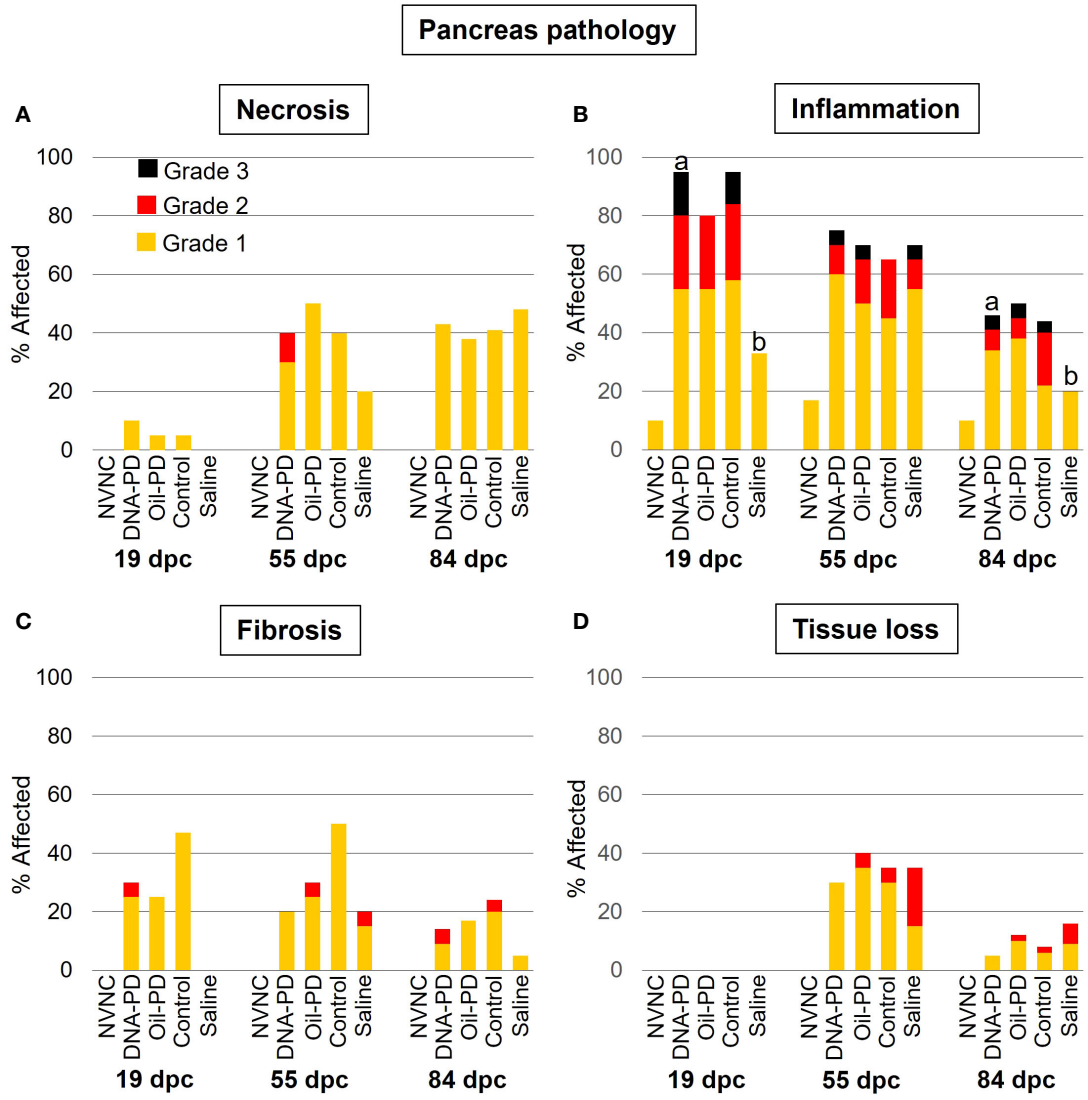
The prevalence and severity of necrosis **(A)**, inflammation **(B)**, fibrosis **(C)** and tissue loss **(D)** of the pancreas sampled at 19 (n=20), 55 (n=20) and 84 (n= 42-49) dpc. NVNC indicates non-vaccinated and non-challenged controls (n=10). Different letters (a, b) denote statistically significant differences between the groups within each pathology criterium and sampling point (Ordinal logistic regression p<0.05) with NVNC excluded. Note that the total height of each bar represents the overall prevalence of each finding.

Very minor degrees of pancreatic necrosis were registered at 19 dpc while low to moderate levels, similar across the groups, were observed at 55 and 84 dpc (**Figure 6A**). The prevalence and severity of pancreatic inflammation peaked at 19 dpc and gradually tapered off over time with significantly lower levels in the Saline group compared to the DNA-PD group at 19 and 84 dpc but not at 55 dpc (**Figure 6B**). The Saline groups also exhibited less fibrosis in the pancreas than the immunized groups, but only at 19 and 84 dpc. Only low to moderate levels of fibrosis were found in the immunized groups with no differences between them (**Figure 6C**). No pancreatic tissue loss was registered at 19 dpc, and moderate loss was observed in the immunized groups at 55 dpc, with the Saline group being the most severely affected. At 84 dpc, all groups displayed a marked reduction in pancreas tissue loss compared to the earlier time point (**Figure 6D**).

**Figure 7:**
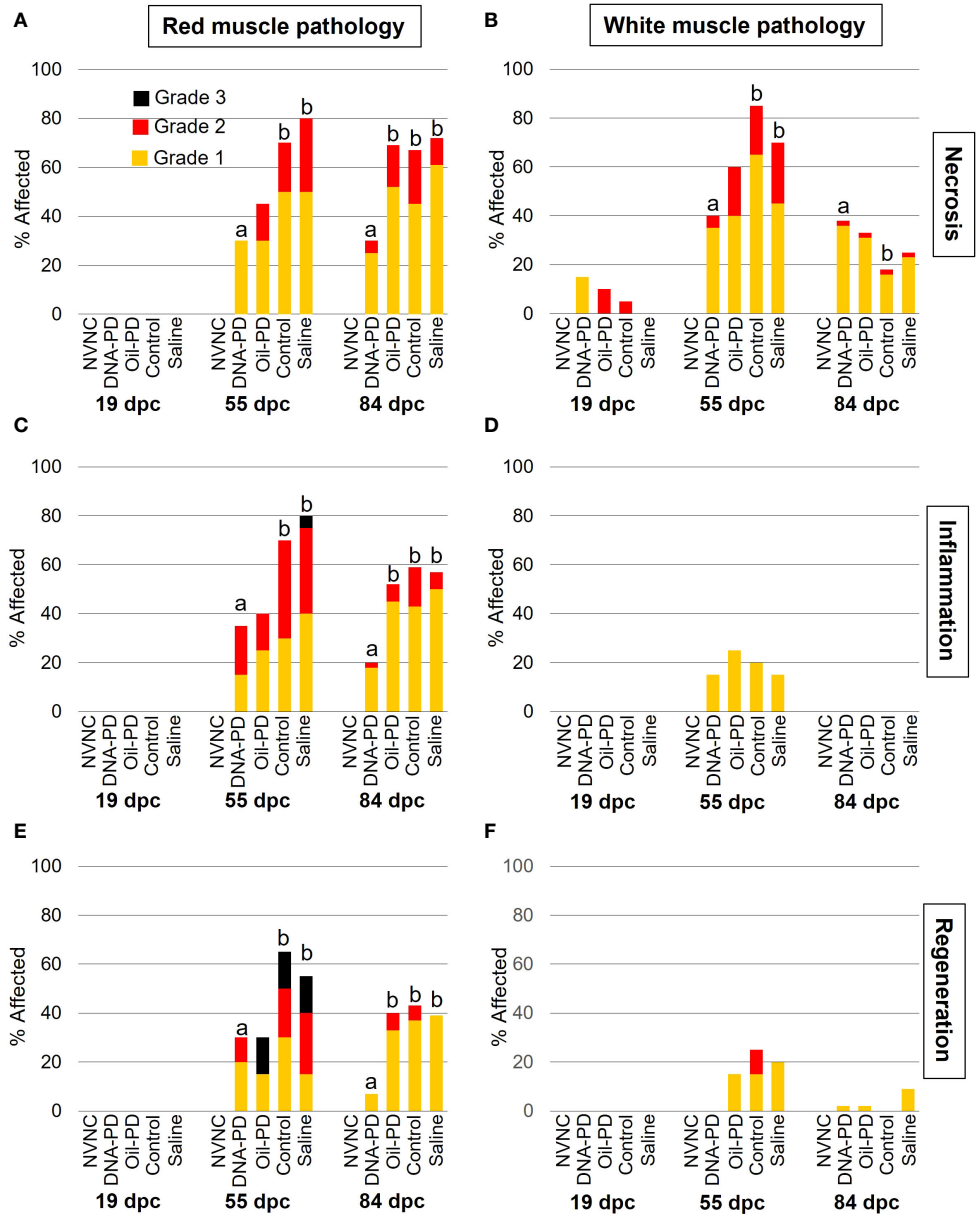
The prevalence and severity of red and white muscle necrosis **(A, B)**, inflammation **(C, D)** and regeneration **(E, F)** sampled at 19 (n=20), 55 (n=20) and 84 (n= 42-49) dpc. NVNC indicates non-vaccinated and non-challenged controls (n=10). Different letters (a, b) denote statistically significant differences between the groups within each pathology criterium and sampling point (Ordinal logistic regression p<0.05) with NVNC excluded. Note that the total height of each bar represents the overall prevalence of each finding.

Except for very minor necrosis in the white muscle, no pathologic changes were found in either muscle type at 19 dpc (**Figure 7**). In general, moderate to severe necrosis, inflammation and regeneration levels were registered in red muscle, peaking at 55 dpc, followed by somewhat reduced levels at 84 dpc. While the DNA-PD group had red muscle pathology scores similar to those of the Oil-PD group at 55 dpc, these were significantly less than those found in the Control and Saline groups. At 84 dpc however, the DNA-PD group had significantly lower red muscle pathology scores than the other fish groups. Prevalence, severity, and statistical differences in the white muscle necrosis between the groups at 55 dpc (**Figure 7B**) corresponded well with those found in the red muscle at the same timepoint (**Figure 7A**). At 84 dpc, the prevalence and severity of necrosis in the white muscle was markedly reduced compared to the 55 dpc levels. In contrast to the 55 and 84 dpc red muscle results, white muscle inflammation and regeneration levels were minor or absent (**Figures 7D, F**).

### 3.3 Transmission study 1 (TS1)

#### 3.3.1 Transmission of SAV from vaccinated fish to naïve cohabitant fish

The concentration of SAV RNA in the water in Tanks 1-10 at 21 dpc was significantly lower in those holding the DNA-PD group compared to those holding the Control (p=0.043) and the Saline groups (p=0.017), but not significant (p=0.080) when compared with the Oil-PD group (**Table 5**). At 21 dpc when the naïve cohabitant fish were added, no SAV RNA was detected in Tanks 1 and 5 (DNA-PD) and 2 (Oil-PD), while Tanks 3 (Control) and 4 (Saline) had 5.8_Log10_ and 4.65_Log10_ SAV RNA copies/L, respectively.

**Table 5:** Concentration of SAV RNA copies (log^10^) per liter of seawater in Tanks 1-10 of the transmission studies containing the challenged treatment groups.

Time point	Group	Log^10^ RNA copies/liter (Tank no.)			Coefficient (95% CI); p-value
21 dpc	DNA-PD	n.d. (1)	n.d. (5)	2.23 (7)^c^	Baseline 0 (-)
	Oil-PD	n.d. (2)	5.42 (6)	2.68 (8)^c^	2.68 (-0.44 − 5.8); 0.080
	Control	5.80 (3)	n.a.	3.66 (9)^c^	3.66 (0.16 − 7.14); 0.043*
	Saline	4.65 (4)	n.a.	4.19 (10)^c^	4.65 (1.16 − 8.14); 0.017*
34 dpc (13 dpe)	DNA-PD	6.03 (1)^a^	2.59 (5)^b^	3.05 (7)^c^	n.a.
	Oil-PD	5.09 (2)^a^	4.08 (6)^b^	5.04 (8)^c^	n.a.
	Control	4.29 (3)^a^	n.a.	4.22 (9)^c^	n.a.
	Saline	5.28 (4)^a^	n.a.	4.87 (10)^c^	n.a.
47 dpc (26 dpe)	DNA-PD	3.24 (1)^a^	2.19 (5)^b^	2.20 (7)^c^	n.a.
	Oil-PD	2.74 (2)^a^	2.20 (6)^b^	4.79 (8)^c^	n.a.
	Control	3.47 (3)^a^	n.a.	2.13 (9)^c^	n.a.
	Saline	2.17 (4)^a^	n.a.	3.29 (10)^c^	n.a.

Results of the median regression model applicable only at the first time point shows the coefficient with 95% confidence intervals (CI) and corresponding p-values.

**^a^** naïve cohabitant fish were added to these tanks at 21 dpc (TS1).

**^b^** similarly immunized cohabitant fish were added to each of these tanks at 21 dpc (TS2). The subsequent sampling points also reflect the days post exposure (dpe) of the cohabitant fish to the challenged fish. ^c^ No cohabitant fish were added to these tanks. “n.d.” = none detected, i.e. below the assay detection limit. “n.a.” = not applicable. “*” denotes significant difference from the DNA-PD group.

**Figure 8:**
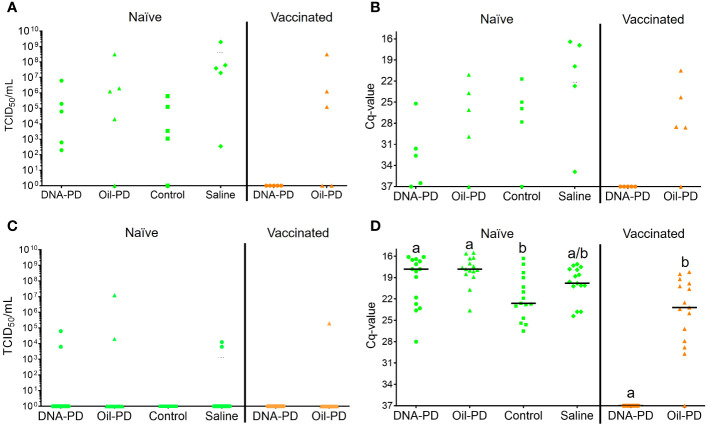
Individual SAV2 viremia shown as TCID_50_ levels **(A, C)** and Cq-values from hearts **(B, D)** of the naïve (green, left, TS1) and vaccinated (orange, right, TS2) cohabitant fish sampled 13 dpe (**(A, B)**; 5 fish/group) and 26 dpe (**(C, D)**; 15 fish/group) with treatment groups (=tanks) indicated on the X-axis. The vaccinated cohabitant fish in each tank had the same vaccination status as their pre-challenged counterparts (their results shown in **Supplementary Figure S6**). The Y-axis value of 37 denotes the negative cut-off value. Statistical analysis was not carried out for the 13 dpe data set due to small sample sizes (n = 5/group). Different letters (a, b) denote significant differences (Median regression analysis p< 0.004).

**Figure 9:**
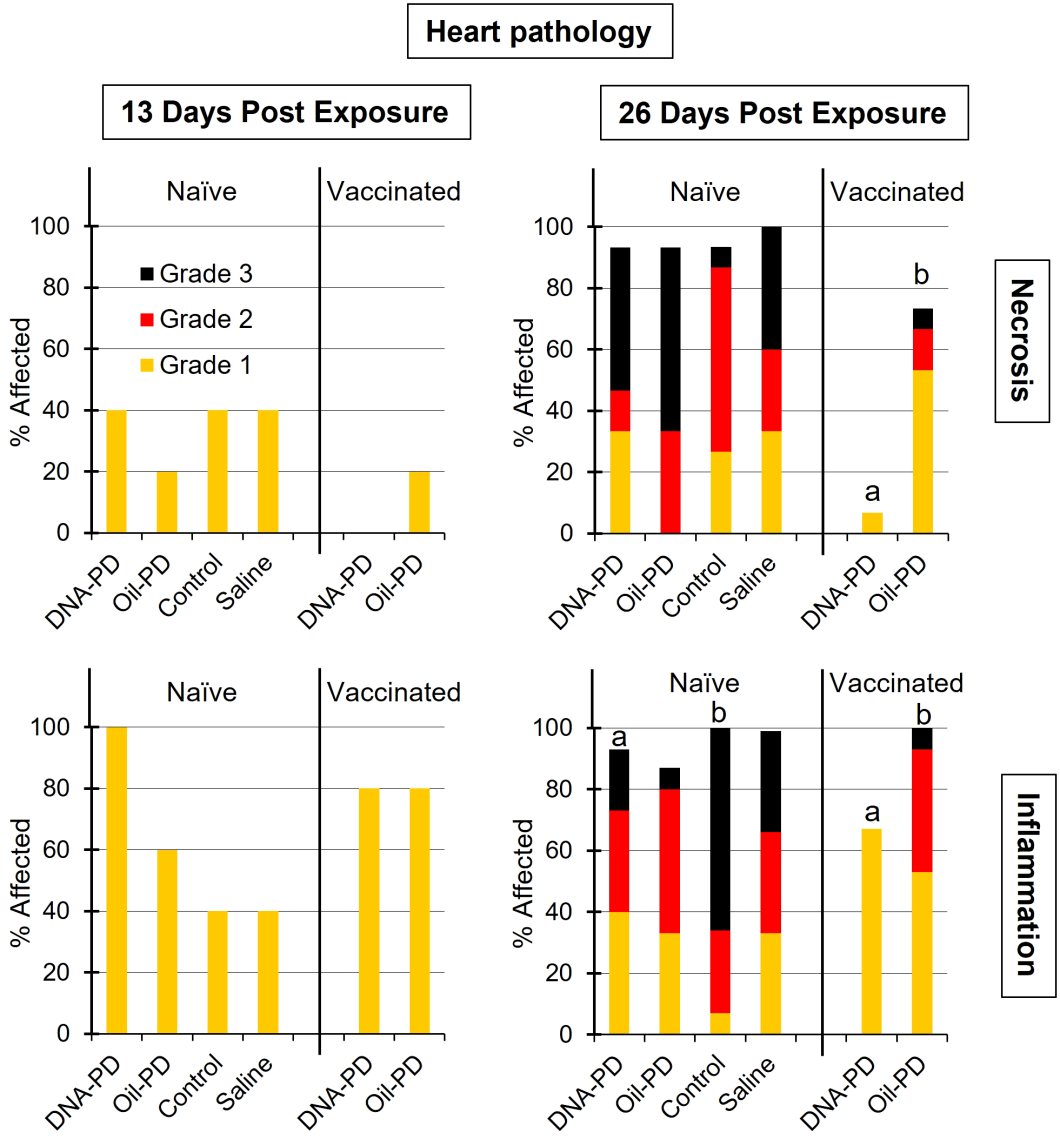
The prevalence and severity of cardiac necrosis and inflammation (regeneration mostly absent - data not shown) of the naïve- (TS1) and vaccinated (TS2) cohabitant fish in each tank, 13 (n=5) and 26 (n=15) days post exposure (dpe). Different letters (a, b) denote significant differences (Ordinal logistic regression p<0.01). Note that the total height of each bar represents the overall prevalence of each finding.

**Figure 10:**
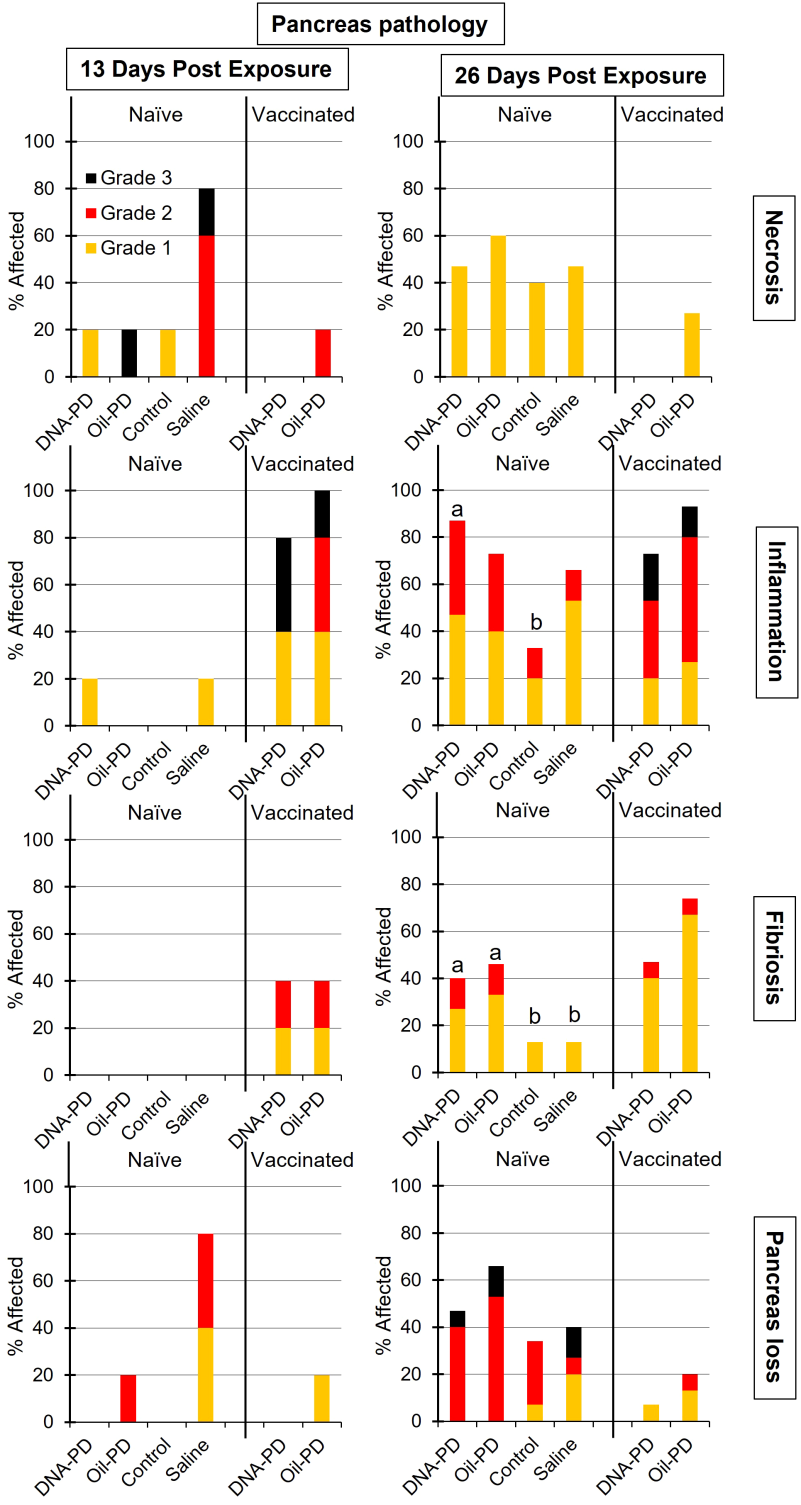
The prevalence and severity of necrosis, inflammation, fibrosis and tissue loss of the pancreas in the naïve- (TS1) and vaccinated (TS2) cohabitant fish in each tank, sampled 13 (n=5) and 26 (n=15) days post exposure (dpe). Different letters (a, b) denote significant differences (Ordinal logistic regression p<0.005). Note that the total height of each bar represents the overall prevalence of each finding.

Thirteen days post exposure (dpe), (13 dpe =34 dpc), of the naïve cohabitants to the pre-challenged groups in Tanks 1-4, high number of copies of SAV RNA/L were measured in the water: ranging from 4.29_Log10_ (Tank 3/Control), 5.09_Log10_ (Tank 2/Oil-PD); 5.28_Log10_ (Tank 4/Saline) and 6.03_Log10_ (Tank 1/DNA-PD, **Table 5**). The viremia and the PCR data from the naïve fish sampled at 13 dpe correspond with ≥4 of 5 fish in each group infected with SAV (**Figures 8A, B**). Between one and two of the 5 naïve fish sampled from each of the 4 tanks/groups at 13 dpe had Grade 1 levels of cardiac necrosis (**Figure 9**). Grade 1 cardiac inflammation levels were also observed at 13 dpe but with increased prevalence in the naïve fish residing with the DNA-PD and Oil-PD groups. The naïve fish residing with the Saline group (4 of 5 fish) displayed more severe necrosis and loss of pancreas tissue at 13 dpe than their counterparts residing with the other groups while pancreas inflammation and fibrosis were largely absent (**Figure 10**).

At 26 dpe (47 dpc), the shedding of SAV RNA into the water had dropped to low levels, ranging from 2.17_Log10_ to 3.47_Log10_ copies/L in Tanks 1-4 that harbored the naïve cohabitants (**Table 5**). This correlated with the observed reduced viremia where only 6 of 60 naïve fish tested were positive (**Figure 8C**). In contrast, the qPCR of the hearts of all naïve fish (TS1) were positive, with significantly more SAV RNA in the hearts of those residing with the DNA-PD and the Oil-PD groups compared to those residing with the Control group but not the Saline group (**Figure 8D**). These results align with more severe heart necrosis observed in the naïve fish residing with the DNA-PD and the Oil-PD groups compared to the fish residing with the Control and the Saline groups (**Figure 9**). At 26 dpe, the severity of cardiac inflammation in the naïve fish was markedly more severe than the 13 dpe levels, and significantly greater in the fish residing with the Control group than those residing with the DNA-PD group (**Figure 9**). In contrast to loss of pancreas tissue, the overall prevalence and severity of inflammation and fibrosis in the pancreas of the naïve fish were markedly greater at 26 dpe compared to the very low levels or absence of these lesions at 13 dpe (**Figure 10**). These combined results confirm the presence of PD in all the naïve cohabitant cohorts irrespective of which pre-challenged treatment group they resided with.

### 3.4 Transmission study 2 (TS2)

#### 3.4.1 Transmission of SAV2 from vaccinated fish to vaccinated cohabitant fish

At 21 dpc (=0 dpe), no SAV RNA was detected in the water of Tank 5 (DNA-PD) while 5.42_Log10_ copies/L were measured in Tank 6 (Oil-PD) just prior to similarly vaccinated cohabitant fish were added to each of these tanks. At 13 dpe, the SAV RNA levels in the water in these tanks measured 2.59_Log10_ copies/L in tank 5 (DNA-PD) and 4.08_Log10_ copies/L in tank 6 (Oil-PD) (**Table 5**). None of the 5 DNA-PD vaccinated cohabitant fish in Tank 5 were viremic or PCR-positive at this timepoint. In contrast, 3/5 of the Oil-PD vaccinated cohabitant fish in Tank 6 were viremic and 4/5 were SAV PCR-positive in heart samples (**Figures 8A, B**). Cardiac necrosis was largely absent in both groups at 13 dpe while low levels of cardiac inflammation (Grade 1) were found in 4 of 5 fish of both groups (**Figure 9**). Whereas a low prevalence of pancreas necrosis was observed in one of the 5 fish in the Oil-PD group, most fish in both groups exhibited inflammation in the pancreas ranging from low (Grade 1) to severe (Grade 3) level. Moderate levels of pancreas fibrosis were also registered in both groups while the pancreas tissue was largely unaffected (**Figure 10**).

At 26 dpe, the SAV RNA levels in the 10 tanks were generally much lower than measured at the earlier timepoints, with ~2.20_Log10_ copies/L in tanks containing the vaccinated cohabitants (**Table 5**). The viremia and PCR results of hearts at 26 dpe resembled those found at 13 dpe with no SAV detected in any of the 15 DNA-PD vaccinated cohabitant fish in Tank 5. In the Oil-PD counterparts in Tank 6, one fish had viremia, and 14 of 15 fish were PCR positive in heart samples with Cq-values ranging from 29.7 to 18.2 and a median of 23.2 (**Figures 8C, D**). Cardiac necrosis especially, and also inflammation levels were markedly lower in the DNA-PD cohabitant fish in Tank 5 than found in the naïve fish groups at 26 dpe in Tanks 1-4, and significantly lower compared to their Oil-PD cohabitant counterparts in Tank 6 (**Figure 9**). Among the vaccinated cohabitants, the DNA-PD group generally exhibited less pancreas necrosis and tissue loss than their Oil-PD equivalents. Like the results at 13 dpe, a high prevalence and severity of inflammation were observed in the pancreas of both the vaccinated cohabitant groups at 26 dpe. At this timepoint, moderate pancreas fibrosis levels were observed in the vaccinated cohabitant groups (**Figure 10**).

Only 4 of the 198 pre-challenged fish remaining in the 10 tanks at 26 dpe (47 dpc) were viremic. The PCR Cq-values of hearts in the pre-challenged groups that resided with the naïve cohabitant fish (Tanks 1-4) were similar. Of these only 6 fish were PCR-negative, 4 of which belonging to the DNA-PD group. In contrast, the DNA-PD group that resided with similarly vaccinated cohabitants (Tank 5) or no cohabitant fish (Tank 7) had markedly reduced Cq-value medians, with 40% (8/20) and 58% (12/19) of the fish being PCR-negative, respectively (**Supplementary Figure S6**). Histopathological evaluation of the heart (**Supplementary Figure S7**) and pancreas (**Supplementary Figure S8**) of the pre-challenged fish groups not containing cohabitants at 47 dpc (Tanks 7-10) revealed similar levels of anomalies as recorded for those organs in the Efficacy study at 55 dpc (**Figures 5**, **6**). At this timepoint, the heart and pancreas of the DNA-PD group residing with the naïve cohabitants (Tank 1) were generally more affected than their counterparts in Tanks 5 and 7 that resided with similarly vaccinated cohabitants or without cohabitants.

## 4 Discussion

### 4.1 Efficacy study

No significant differences in weight were found between the treatment groups at either 19 or 55 dpc, while at 84 dpc, the DNA-PD group had significantly outgrown the other groups. With no differences in weight or i.p. side effect scores registered between the immunized groups before the challenge, the significantly higher weight of the DNA-PD group at 84 dpc was likely caused by the difference in protection against PD-associated growth loss as found in similar experiments for SAV3 (11, 12). This finding aligns with the qPCR results of the hearts at 84 dpc with the DNA-PD group harboring significantly less SAV RNA than the other groups, while high and similar levels were measured in all the groups at 55 dpc. Lower cardiac histopathology scores in the DNA-PD group at 84 dpc indicate less impact on heart health in this group. The pathologies of the pancreas at this timepoint were, in contrast, very similar between the groups and markedly milder than found in similar SAV3 studies. Based on the above, the effect against PD-associated growth loss and clearance of SAV2 RNA was significantly higher in the PD-DNA group than the other groups.

Cumulative mortality was low and similar across treatment groups (9.1 to 12.6%) although time to death was notably delayed in the DNA-PD group compared to the other treatment groups. The Cq-values in the hearts of the fish that died between 27 and 53 dpc showed markedly less SAV RNA in the DNA-PD group compared to the other groups. While the underlying mechanisms have not been studied, it can be speculated that these findings result from a higher vaccine efficacy in the DNA-PD group, as supported by the comparative growth and histopathology results. Why the end-point mortality in the Saline group was lower than the Oil-PD and the Control groups is puzzling. The cumulative mortality in the Saline, Oil-PD and Control groups was similar until about 40 dpc, after which the mortality curve for the Saline group flattened while the mortality of the Oil-PD and Control groups was still risimg. There were high SAV2 neutralization titers in all groups at 84 dpc, and one possibility is that the immunity induced by the challenge virus occurred earlier in the naïve Saline group than in the Oil-PD and Control groups. Still, additional time-course studies would be required to understand this.

The SAV2 challenge isolate employed in this study was previously used in a similar challenge model shown to cause markedly less mortality compared to SAV3 (7). Therefore, the mortality levels found in the Efficacy study were higher than expected, and similar to those found in previous vaccine efficacy studies using a SAV3 isolate and the same cohabitation challenge model (11, 12) as employed in this study. In contrast to these studies including a SAV3 challenge, in which 96% (134/140) of the fish were viremic at 19 dpc, only 16% (13/80) were viremic at 19 dpc in the present study. Only 3 hearts of the 13 viremia-positive fish were PCR-positive. This finding agrees with results from an earlier SAV3 infection study demonstrating viremia appearing for a short period before any viral RNA can be detected in the hearts (41). The delay in the onset of the SAV2 infection compared to the aforementioned SAV3 studies is likely due to lower virulence of the SAV2 as previously reported for this genotype (7), as also seen during field outbreaks in Norway (42). Further, the challenge dose each of the shedder fish received in this study was considerably lower (3.52 x 10^3^ TCID_50_) than that used in the SAV3 studies (1.26 x 10^4^ TCID_50_). This may also have contributed to the delayed onset of the SAV2 infection compared to SAV3 challenges (11, 12).

With 94% (75/80) of the fish hearts positive for SAV by PCR at 55 dpc, and after that reductions in the SAV RNA in all the groups at 84 dpc, suggests that the peak in the viremic phase occurred between 19 and 55 dpc. This assumption is supported by the absence of heart and pancreas necrosis and pancreas tissue loss at 19 dpc. Additional evidence supporting this assumption includes the degree of the heart regeneration peaking at 55 dpc and the 85% (67/79) prevalence of PCR-positive heart samples collected in the transmission studies at 47 dpc from all the fish groups (Tanks 7-10) without any cohabitant fish added.

The histopathological changes of the heart and pancreas induced by SAV2 at 55 and 84 dpc were much milder than those registered in the previous SAV3 studies (11, 12). At the same time, the lesion scores in red and white muscles were similar to what was observed in this study. Interestingly, the loss of pancreas tissue at 55 dpc was mostly Grade 1 levels and present in ≤40% of the samples from all groups. At 84 dpc, the pancreas tissue loss scores were reduced by more than half compared to 55 dpc. This is in stark contrast to the markedly more severe and progressive development of pancreas tissue loss in the earlier SAV3 studies. Loss of pancreatic tissue has been suggested to be an important factor causing the growth loss associated with PD (11, 12). This may explain why the growth difference between the DNA-PD and the other groups at 84 dpc, albeit significant, is not as large as in similar treatment groups in earlier SAV3 efficacy studies. This further supports previous studies reporting SAV2 as less virulent than SAV3 (7, 42).

### 4.2 Transmission studies

Despite a low prevalence of detectable SAV2 infection in the challenge Tank A at 19 dpc in 3 of the 4 treatment groups, all the pre-challenged fish groups residing in the 10 tanks were infected and developed varying levels of changes in internal organs typical of PD during the 26-day transmission studies. At 21 dpc, water in 7 of the 10 tanks contained SAV RNA ranging from 2.23_Log10_ to 5.8_Log10_ copies/L. At this timepoint, the levels of SAV RNA in the water containing the DNA-PD group were below the detection limit (0) in two of the tanks (1 and 5) and very low (170 copies/L) in tank 7. These levels were significantly lower than measured in the tanks holding the Control and Saline groups while not significant (p=0.08) compared to the Oil-PD group.

At 34 dpc (=13 dpe), SAV RNA was detected in the water of all 10 tanks, with similar levels (4.08_Log10_ to 5.28_Log10_ copies/L) within and between the replicate tanks containing the Oil-PD, Control and Saline groups. The corresponding levels for the DNA-PD group were, in contrast, highly variable ranging from 6.03_Log10_ in Tank 1 (including naïve cohabitants, TS1) to 2.59_Log10_ and 3.05_Log10_ SAV RNA copies/L in Tanks 5 (with immunized cohabitants, TS2) and 7 (without any cohabitants), respectively. Of the 20 naïve cohabitant fish sampled from Tanks 1-4 (5 fish/tank, TS1) at 13 dpe, 18 were viremic and 17 with PCR-positive hearts. The results of the heart and pancreas pathologies confirm shedding and effective transmission of the virus to naïve fish in Tanks 1-4 irrespective of the vaccination status of the pre-challenged fish groups.

Of the 5 DNA-PD vaccinated cohabitant fish in Tank 5 (TS2) sampled at 13 dpe, none were viremic, their hearts negative by PCR and key histopathology indicators (pancreas necrosis, tissue loss and heart necrosis) largely absent. Of the 5 Oil-PD cohabitant fish in Tank 6 sampled at 13 dpe (TS2), most were both viremic (3/5), with PCR-positive hearts (4/5) and key histopathological indicator showing early onset of PD. These findings corroborate with lower virus levels in water in tanks containing the DNA-PD group (2.59_Log10_ and 3.05_Log10_ RNA copies/L), indicative of less viral shedding, concomitantly with PD-DNA vaccinated cohabitant fish being better protected against viral challenge, or a combination of these factors.

At 47 dpc (=26 dpe), SAV RNA was again detected in the water of all 10 tanks, but at much lower levels than the earlier timepoint, suggesting that the viremic phases had largely culminated in all fish groups. This finding is supported by the extensive reduction in prevalence of viremia in the naïve cohabitants in Tanks 1-4 (TS1) from 95% (19/20) at 13 dpe to 10% (6/60) at 26 dpe. In contrast, all the hearts of the 60 naïve fish sampled at 26 dpe were PCR positive with Cq-levels ≤28. Of these, the SAV RNA levels in the Control group were significantly lower than those measured in the DNA-PD and the Oil-PD groups, but not the Saline group. The reason for this finding remains unknown but may be due to varying kinetics of this parameter between groups at given timepoints. However, the prevalences of heart necrosis, pancreas necrosis and loss of pancreas tissue at 26 dpe in the naïve cohabitant cohorts of >90%, ≥40% and >30%, respectively, confirms the successful transmission of PD to these cohorts irrespective of the immune status of the ‘donor group’.

Of the immunized cohabitant fish sampled 26 dpe (47 dpc), the virus was cultured from 7% (1/15) of the fish in the Oil-PD group, while no viremic fish were found from the DNA-PD group. The PCR results from the hearts of these two groups revealed a highly significant difference. The fish in the DNA-PD group were all PCR-negative while 93% (14/15) of the hearts of the Oil-PD group were PCR-positive with Cq-values ranging from 30 to 18 with a median of 23. When coupled with the heart necrosis, pancreas necrosis and loss of pancreas tissue largely absent or low in the DNA-PD immunized cohabitants at 26 dpe (**Figures 9**, **10**), the results demonstrate that these cohorts, in contrast to their Oil-PD counterparts, effectively resisted against the SAV2 challenge pressure endured in Tank 5. The discrepancy between virus culture-positive and PCR-positive fish in the Oil-PD group might align with an understanding that SAV forms defective viral genomes during replication *in vivo* (43, 44).

When compared to the PD levels that developed in the naïve cohabitants residing with the pre-challenged DNA-PD (Tank 1), the results signify the value of ‘herd immunity’ in the aquatic environment when employing efficacious vaccines which can inhibit disease transmission and thus, curb the spread of an epizootic.

Water was sampled at three timepoints to quantify SAV RNA shed by the different treatment groups. As such, these measurements offer only snapshot overviews, limiting insights into the dynamic process of viremia that may vary with time based on the relative protection level provided by the vaccine. Future efforts should consider more frequent analysis of SAV RNA in water to better understand the viremia phase associated with PD.

The results of the Efficacy and transmission studies are complementary, demonstrating that the DNA vaccine employed significantly reduces the impact of waterborne SAV2 infection in the presence of susceptible cohorts and further inhibits the spreading of the virus when the neighboring cohorts have been immunized with the same vaccine. Conversely, the fish in the Oil-PD group neither demonstrated significant protection against PD, nor the capacity to curb further transmission of SAV2 to similarly vaccinated cohabitant fish.

## 5 Conclusions

Except for the mortality levels, the results presented here align with previous studies showing SAV2 less virulent than SAV3. In the Efficacy study, the DNA-PD group was significantly protected against infection-induced growth inhibition and pathology, particularly in the heart and muscle tissues. In contrast, the pancreas pathologies were markedly less severe than observed in similar SAV3 studies. While at 55 dpc, most of the fish in all the groups were PCR positive with similar Cq-value profiles, the 84 dpc SAV RNA levels in the heart were significantly reduced in the DNA-PD group compared to the other groups indicating more effective clearance of the infection.

In the transmission study, the DNA-PD group shed SAV2 at significantly reduced levels at 21 dpc compared to the Control and Saline groups but not the Oil-PD group. All the pre-challenged groups transmitted SAV2 effectively to their naïve cohabitant fish (TS1). In contrast, the DNA-PD vaccinated cohabitants remained free from SAV2 infection and PD-related pathologies while exposed to their pre-challenged and similarly vaccinated counterparts (TS2). These results are likely caused by the improved protective immunity in this group and lower challenge levels from the pre-challenged DNA-PD vaccinated fish. These results signify how ‘herd immunity’, when achieved by the use of an efficacious vaccine, can curb transmission and help limit the geographic spreading of waterborne diseases such as PD.

## Data availability statement

The original contributions presented in the study are included in the article/**Supplementary Material**. Further inquiries can be directed to the corresponding author.

## Ethics statement

The animal studies were approved by Norwegian Animal Research Authority (FOTS ID, 27332). The studies were conducted in accordance with the local legislation and institutional requirements. Written informed consent was obtained from the owners for the participation of their animals in this study.

## Author contributions

RT: Conceptualization, Data curation, Investigation, Supervision, Writing – original draft, Validation, Visualization. AR: Data curation, Investigation, Project administration, Supervision, Writing – review & editing. JW: Data curation, Formal analysis, Investigation, Methodology, Validation, Writing – review & editing. HS: Data curation, Investigation, Methodology, Supervision, Validation, Writing – review & editing. ES: Data curation, Formal analysis, Methodology, Validation, Writing – review & editing. ER: Conceptualization, Methodology, Writing – review & editing. ØE: Conceptualization, Methodology, Visualization, Writing – review & editing. JR: Funding acquisition, Project administration, Resources, Supervision, Visualization, Writing – review & editing.

## References

[1] Jansen MD Bang Jensen B McLoughlin MF Rodger HD Taksdal T Sindre H. The epidemiology of pancreas disease in salmonid aquaculture: a summary of the current state of knowledge. J Fish Dis. (2017) 40:141–55. doi: 10.1111/jfd.12478 27136332

[2] Weston JH Welsh MD McLoughlin MF Todd D. Salmon pancreas disease virus, an alphavirus infecting farmed Atlantic salmon, Salmo salar L. Virology. (1999) 256:188–95. doi: 10.1006/viro.1999.9654 10191183

[3] Weston J Villoing S Bremont M Castric J Pfeffer M Jewhurst V. Comparison of two aquatic alphaviruses, salmon pancreas disease virus and sleeping disease virus, by using genome sequence analysis, monoclonal reactivity, and cross-infection. J Virol. (2002) 76:6155–63. doi: 10.1128/JVI.76.12.6155-6163.2002 PMC136221 12021349

[4] Graham D. Prospective longitudinal studies of salmonid alphavirus infections on two Atlantic salmon farms in Ireland; evidence for viral persistence. J Fish Dis. (2010) 33:623. doi: 10.1111/j.1365-2761.2009.01096.x 19732268

[5] Jansen MD Wasmuth MA Olsen AB Gjerset B Modahl I Breck O. Pancreas disease (PD) in sea-reared Atlantic salmon, Salmo salar L., in Norway; a prospective, longitudinal study of disease development and agreement between diagnostic test results. J Fish Dis. (2010) 33:723–36. doi: 10.1111/j.1365-2761.2010.01176.x 20609035

[6] Bang Jensen B Kristoffersen AB Myr C Brun E. Cohort study of effect of vaccination on pancreas disease in Norwegian salmon aquaculture. Dis Aquat Organ. (2012) 102:23–31. doi: 10.3354/dao02529 23209075

[7] Taksdal T Jensen BB Bockerman I McLoughlin MF Hjortaas MJ Ramstad A. Mortality and weight loss of Atlantic salmon, Salmon salar L., experimentally infected with salmonid alphavirus subtype 2 and subtype 3 isolates from Norway. J Fish Dis. (2015) 38:1047–61. doi: 10.1111/jfd.12312 25322679

[8] Taksdal T Wiik-Nielsen J Birkeland S Dalgaard P Morkore T. Quality of raw and smoked fillets from clinically healthy Atlantic salmon, Salmo salar L., following an outbreak of pancreas disease (PD). J Fish Dis. (2012) 35:897–906. doi: 10.1111/j.1365-2761.2012.01428.x 22924617

[9] McLoughlin MF Graham DA Norris A Matthews D Foyle L Rowley HM. Virological, serological and histopathological evaluation of fish strain susceptibility to experimental infection with salmonid alphavirus. Dis Aquat Organ. (2006) 72:125–33. doi: 10.3354/dao072125 17140135

[10] McLoughlin MF Nelson RN McCormick JI Rowley HM Bryson DB. Clinical and histopathological features of naturally occurring pancreas disease in farmed Atlantic salmon, Salmo salar L. J Fish Dis. (2002) 25:33–43. doi: 10.1046/j.1365-2761.2002.00334.x

[11] Thorarinsson R Wolf JC Inami M Phillips L Jones G Macdonald AM. Effect of a novel DNA vaccine against pancreas disease caused by salmonid alphavirus subtype 3 in Atlantic salmon (Salmo salar). Fish Shellfish Immunol. (2021) 108:116–26. doi: 10.1016/j.fsi.2020.12.002 33285168

[12] Thorarinsson R Wolf JC Inami M Sindre H Skjerve E Evensen Ø.. Effects of a DNA and multivalent oil-adjuvanted vaccines against pancreas disease in Atlantic salmon (Salmo salar) challenged with salmonid alphavirus subtype 3. Fish shellfish Immunol Rep. (2022) 3:100063. doi: 10.1016/j.fsirep.2022.100063 36419608 PMC9680106

[13] Fringuelli E Rowley HM Wilson JC Hunter R Rodger H Graham DA. Phylogenetic analyses and molecular epidemiology of European salmonid alphaviruses (SAV) based on partial E2 and nsP3 gene nucleotide sequences. J Fish Dis. (2008) 31:811–23. doi: 10.1111/j.1365-2761.2008.00944.x 18681902

[14] Tighe AJ Gallagher MD Carlsson J Matejusova I Swords F Macqueen DJ. Nanopore whole genome sequencing and partitioned phylogenetic analysis supports a new salmonid alphavirus genotype (SAV7). Dis Aquat organisms. (2020) 142:203–11. doi: 10.3354/dao03546 33331288

[15] Graham DA Rowley HR Frost P. Cross-neutralization studies with salmonid alphavirus subtype 1-6 strains: results with sera from experimental studies and natural infections. J Fish Dis. (2014) 37:683–91. doi: 10.1111/jfd.12167 23957811

[16] Karlsen M Gjerset B Hansen T Rambaut A. Multiple introductions of salmonid alphavirus from a wild reservoir have caused independent and self-sustainable epizootics in aquaculture. J Gen Virol. (2014) 95:52–9. doi: 10.1099/vir.0.057455-0 24062534

[17] Hjortaas MJ Jensen BB Taksdal T Olsen AB Lillehaug A Trettenes E. Genetic characterization of salmonid alphavirus in Norway. J Fish Dis. (2016) 39:249–57. doi: 10.1111/jfd.12353 25683753

[18] Hjortaas MJ Skjelstad HR Taksdal T Olsen AB Johansen R Bang-Jensen B. The first detections of subtype 2-related salmonid alphavirus (SAV2) in Atlantic salmon, Salmo salar L., in Norway. J Fish Dis. (2013) 36:71–4. doi: 10.1111/j.1365-2761.2012.01445.x 22943794

[19] Sommerset I Wiik Nielsen JO Moldal T VHS Bornø. G Haukaas A Brun E. Fish Health Report 2022. Norwegian: Norwegian Veterinary Institute (2023).

[20] Viljugrein H Staalstrom A Molvaelr J Urke HA Jansen PA. Integration of hydrodynamics into a statistical model on the spread of pancreas disease (PD) in salmon farming. Dis Aquat Organ. (2009) 88:35–44. doi: 10.3354/dao02151 20183963

[21] Kristoffersen AB Viljugrein H Kongtorp RT Brun E Jansen PA. Risk factors for pancreas disease (PD) outbreaks in farmed Atlantic salmon and rainbow trout in Norway during 2003-2007. Prev Vet Med. (2009) 90:127–36. doi: 10.1016/j.prevetmed.2009.04.003 19419787

[22] Aldrin M Storvik B Frigessi A Viljugrein H Jansen PA. A stochastic model for the assessment of the transmission pathways of heart and skeleton muscle inflammation, pancreas disease and infectious salmon anaemia in marine fish farms in Norway. Prev Vet Med. (2010) 93:51–61. doi: 10.1016/j.prevetmed.2009.09.010 19811843

[23] Tavornpanich S Paul M Viljugrein H Abrial D Jimenez D Brun E. Risk map and spatial determinants of pancreas disease in the marine phase of Norwegian Atlantic salmon farming sites. BMC Veterinary Res. (2012) 8:172. doi: 10.1186/1746-6148-8-172 PMC3514396 23006469

[24] Stene A Viljugrein H Yndestad H Tavornpanich S Skjerve E. Transmission dynamics of pancreas disease (PD) in a Norwegian fjord: aspects of water transport, contact networks and infection pressure among salmon farms. J Fish Dis. (2014) 37:123–34. doi: 10.1111/jfd.12090 23452114

[25] Long A Richard J Hawley L LaPatra S Garver K. Transmission potential of infectious hematopoietic necrosis virus in APEX-IHN®-vaccinated Atlantic salmon. Dis Aquat organisms. (2017) 122:213–21. doi: 10.3354/dao03076 28117300

[26] Weli SC Bernhardt L-S Qviller L Myrmel M Lillehaug A. Development and evaluation of a method for concentration and detection of salmonid alphavirus from seawater. J Virological Methods. (2021) 287. doi: 10.1016/j.jviromet.2020.113990 33035567

[27] Bernhardt L Myrmel M Lillehaug A Qviller L Chioma Weli S. Concentration and detection of salmonid alphavirus in seawater during a post-smolt salmon (Salmo salar) cohabitant challenge. Dis Aquat organisms. (2021) 144:61–73. doi: 10.3354/dao03572 33764314

[28] Romstad AB Reitan LJ Midtlyng P Gravningen K Evensen O. Development of an antibody ELISA for potency testing of furunculosis (Aeromonas salmonicida subsp salmonicida) vaccines in Atlantic salmon (Salmo salar L). Biologicals. (2012) 40:67–71. doi: 10.1016/j.biologicals.2011.09.011 22000732

[29] Erdal JI Reitan LJ. Immune response and protective immunity after vaccination of Atlantic salmon (Salmo salar L.) against furunculosis. Fish Shellfish Immunol. (1992) 2:99–108. doi: 10.1016/S1050-4648(05)80039-7

[30] Graham DA Jewhurst VA Rowley HM McLoughlin MF Todd D. A rapid immunoperoxidase-based virus neutralization assay for salmonid alphavirus used for a serological survey in Northern Ireland. J Fish Dis. (2003) 26:407–13. doi: 10.1046/j.1365-2761.2003.00472.x 12946010

[31] Falk K Namork E Dannevig BH. Characterization and applications of a monoclonal antibody against infectious salmon anaemia virus. Dis Aquat Organ. (1998) 34:77–85. doi: 10.3354/dao034077 9828403

[32] Merour E Lamoureux A Biacchesi S Bremont M. Fine mapping of a salmonid E2 alphavirus neutralizing epitope. J Gen Virol. (2016) 97:893–900. doi: 10.1099/jgv.0.000411 26801972

[33] Mikalsen AB Sindre H Mjaaland S Rimstad E. Expression, antigenicity and studies on cell receptor binding of the hemagglutinin of infectious salmon anemia virus. Arch Virol. (2005) 150:1621–37. doi: 10.1007/s00705-005-0502-4 15824888

[34] Midtlyng PJ. Experimental studies on the efficacy and side-effects of intraperitoneal vaccination of Atlantic salmon (Salmo salar L.) against furunculosis. Fish Shellfish Immunol (6). (1996) 335–50. doi: 10.1006/fsim.1996.0034

[35] Hodneland K Endresen C. Sensitive and specific detection of Salmonid alphavirus using real-time PCR (TaqMan). J Virol Methods. (2006) 131:184–92. doi: 10.1016/j.jviromet.2005.08.012 16202457

[36] Jewhurst VA Todd D Rowley HM Walker IW Weston JH McLoughlin MF. Detection and antigenic characterization of salmonid alphavirus isolates from sera obtained from farmed Atlantic salmon, Salmo salar L., and farmed rainbow trout, Oncorhynchus mykiss (Walbaum). J Fish Dis. (2004) 27:143–9. doi: 10.1111/j.1365-2761.2004.00530.x 15009240

[37] Kärber G. Beitrag zur kollektiven Behandlung pharmakologischer Reihenversuche. Naunyn-Schmiedebergs Archiv für experimentelle Pathologie und Pharmakologie. (1931) 162:480–3. doi: 10.1007/BF01863914

[38] Jenberie S Peñaranda MMD Thim HL Styrvold MB Strandskog G Jørgensen JB. Salmonid alphavirus subtype 3 induces prolonged local B cell responses in atlantic salmon (Salmo salar) after intraperitoneal infection. Front Immunol. (2020) 11:1682. doi: 10.3389/fimmu.2020.01682 33013821 PMC7511533

[39] Graham DA Fringuelli E Wilson C Rowley HM Brown A Rodger H. Prospective longitudinal studies of salmonid alphavirus infections on two Atlantic salmon farms in Ireland; evidence for viral persistence. J Fish Dis. (2010) 33:123–35. doi: 10.1111/j.1365-2761.2009.01096.x 19732268

[40] Teige LH Aksnes I Røsæg MV Jensen I Jørgensen JB Sindre H. Detection of specific Atlantic salmon antibodies against salmonid alphavirus using a bead-based immunoassay. Fish Shellfish Immunol. (2020) 106:374–83. doi: 10.1016/j.fsi.2020.07.055 32738513

[41] Jarungsriapisit J Moore LJ Maehle S Skar C Einen AC Fiksdal IU. Relationship between viral dose and outcome of infection in Atlantic salmon, Salmo salar L., post-smolts bath-challenged with salmonid alphavirus subtype 3. Vet Res. (2016) 47:102. doi: 10.1186/s13567-016-0385-2 27760562 PMC5069985

[42] Jansen MD Jensen BB Brun E. Clinical manifestations of pancreas disease outbreaks in Norwegian marine salmon farming - variations due to salmonid alphavirus subtype. J Fish Dis. (2015) 38:343–53. doi: 10.1111/jfd.12238 24661057

[43] Petterson E Guo TC Evensen O Mikalsen AB. Experimental piscine alphavirus RNA recombination in *vivo* yields both viable virus and defective viral RNA. Sci Rep. (2016) 6:36317. doi: 10.1038/srep36317 27805034 PMC5090867

[44] Petterson E Stormoen M Evensen O Mikalsen AB Haugland O. Natural infection of Atlantic salmon (Salmo salar L.) with salmonid alphavirus 3 generates numerous viral deletion mutants. J Gen Virol. (2013) 94:1945–54. doi: 10.1099/vir.0.052563-0 23704276

