# Supplementary material for: Effect of pancreas disease vaccines on infection levels and virus transmission in Atlantic salmon (Salmo salar) challenged with salmonid alphavirus, genotype 2
Source: Front Immunol. 2024 Mar 7;15:1342816. doi: 10.3389/fimmu.2024.1342816 (PMC10955579; doi:10.3389/fimmu.2024.1342816)
Supplement: Supplementary file 1 [file DataSheet_1.zip › Supplementary Figure 1.DOCX]

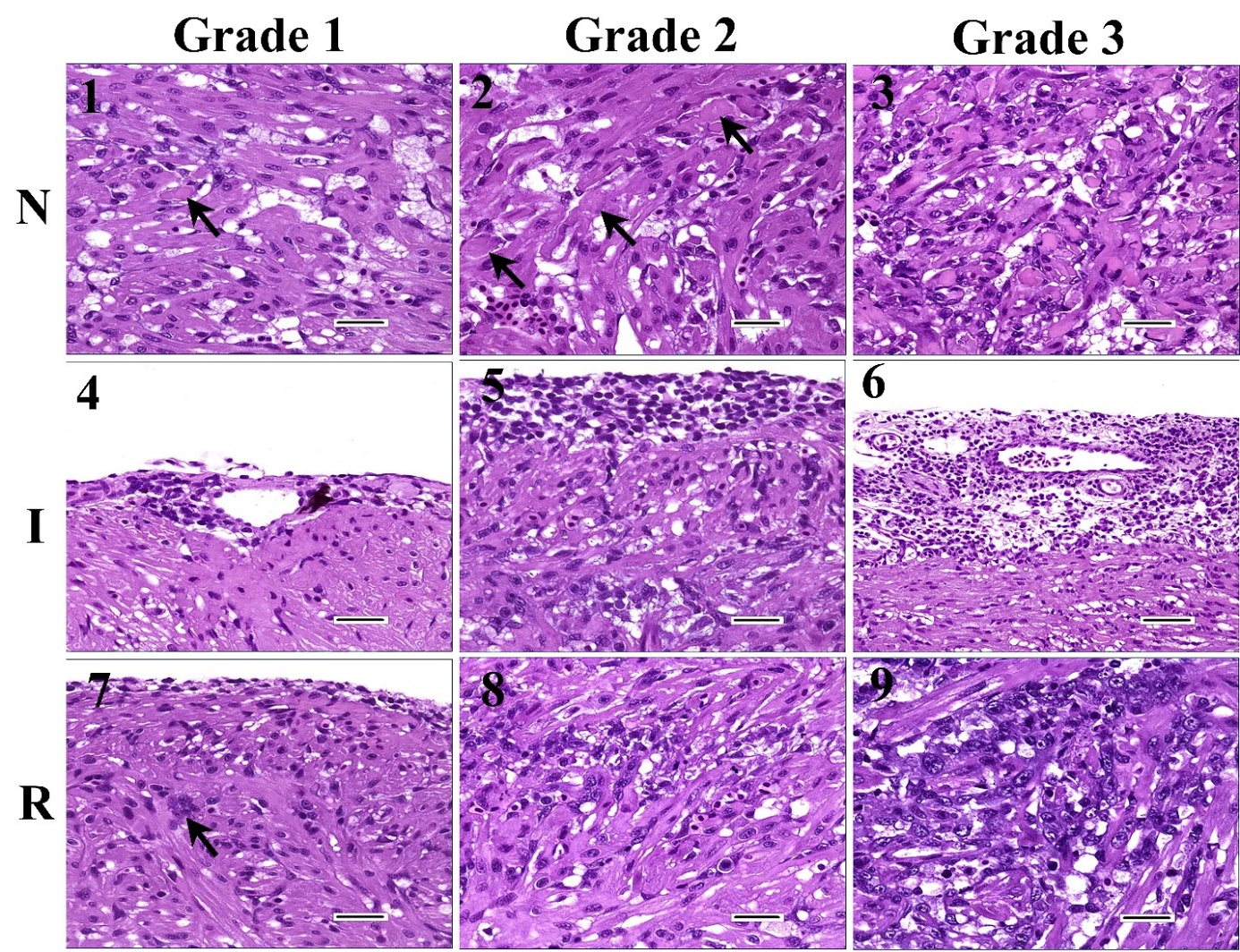


**Figure S1** Severity grading examples of heart findings. 1) Arrow indicates a single small pale irregularly-shaped necrotic myocyte in the cardiac ventricle. 2) Arrows indicate several necrotic myocytes within a high magnification field. 3) The vast majority of myocytes throughout the ventricle are necrotic. 4) There is a small focus of ventricular epicardial mononuclear cell infiltrates. 5) Epicardial infiltrates form a discontinuous layer approximately 3-9 cells deep. 6) Epicardial infiltrates form a continuous layer, frequently 10-20 cells deep, and myocardial infiltrates are also common. 7) Arrow indicates a single focus of regenerating cardiac myocytes, observed as a small cluster of large basophilic nuclei, within the ventricle at the interface between the stratum compactum and stratum spongiosum. 8) Grade 2 is characterized by multiple focally extensive areas of myocyte regeneration. 9) Myocyte regeneration is widespread throughout the ventricle. N = necrosis, I = inflammation, R = regeneration. Bar sizes: images 1-2, 4-5, and 7-9, bar = 25 mm; images 3 and 6, bar = 50 mm.
