# Supplementary material for: Effect of pancreas disease vaccines on infection levels and virus transmission in Atlantic salmon (Salmo salar) challenged with salmonid alphavirus, genotype 2
Source: Front Immunol. 2024 Mar 7;15:1342816. doi: 10.3389/fimmu.2024.1342816 (PMC10955579; doi:10.3389/fimmu.2024.1342816)
Supplement: Supplementary file 1 [file DataSheet_1.zip › Supplementary Figure 2.DOCX]

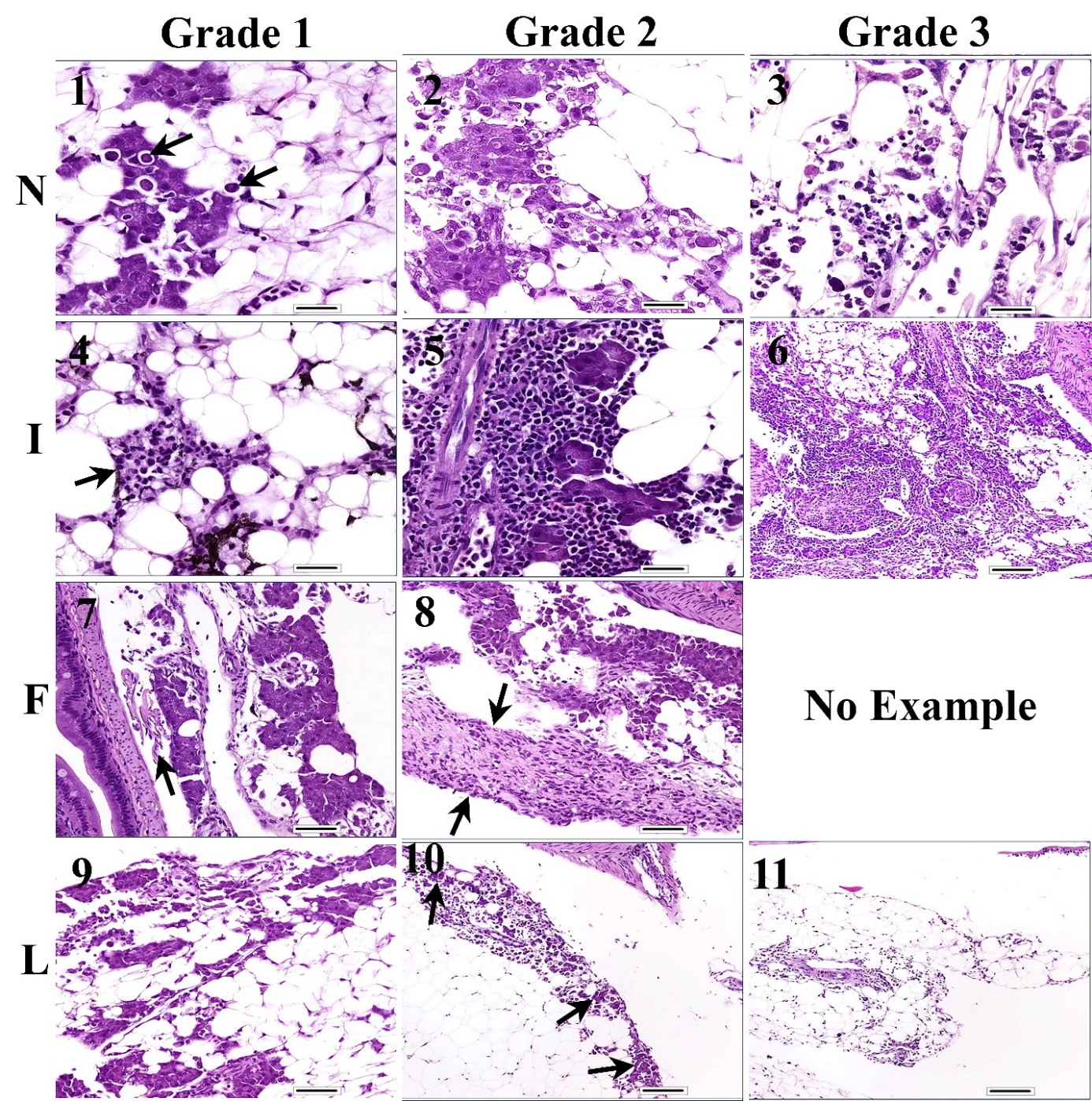


**Figure S2.** Severity grading examples of exocrine pancreas findings. 1) Arrows indicate single cell necrosis (apoptotic-type) of acinar cells. 2) Necrosis is more widespread, but some of the acinar tissue is spared. 3) Essentially all the acinar tissue is necrotic. 4) A small focus of mononuclear cell infiltrates resides within the peri-pancreatic adipose tissue. 5) Focally extensive area of mononuclear cell infiltration. 6) Widespread inflammation involving the pancreas and adjacent mesenteric adipose tissue. 7) A small focus of several immature collagenous fibers. 8) Larger patchy area of immature fibrous connective tissue (Grade 3 fibrosis was not observed in this study). 9) More than half the exocrine pancreas remains, the rest is necrotic. 10) Less than half the exocrine pancreas remains, but some intact acini (arrows) are still evident. 11) No intact acinar tissue remains. N = necrosis, I = inflammation, F = fibrosis, L = loss of acinar tissue. Bar sizes: images 1-5, bar = 25 mm; images 7-9, bar = 50 mm; images 6 and 10-11, bar = 100 mm.
