# Supplementary material for: Effect of pancreas disease vaccines on infection levels and virus transmission in Atlantic salmon (Salmo salar) challenged with salmonid alphavirus, genotype 2
Source: Front Immunol. 2024 Mar 7;15:1342816. doi: 10.3389/fimmu.2024.1342816 (PMC10955579; doi:10.3389/fimmu.2024.1342816)
Supplement: Supplementary file 1 [file DataSheet_1.zip › Supplementary Figure 3.DOCX]

**
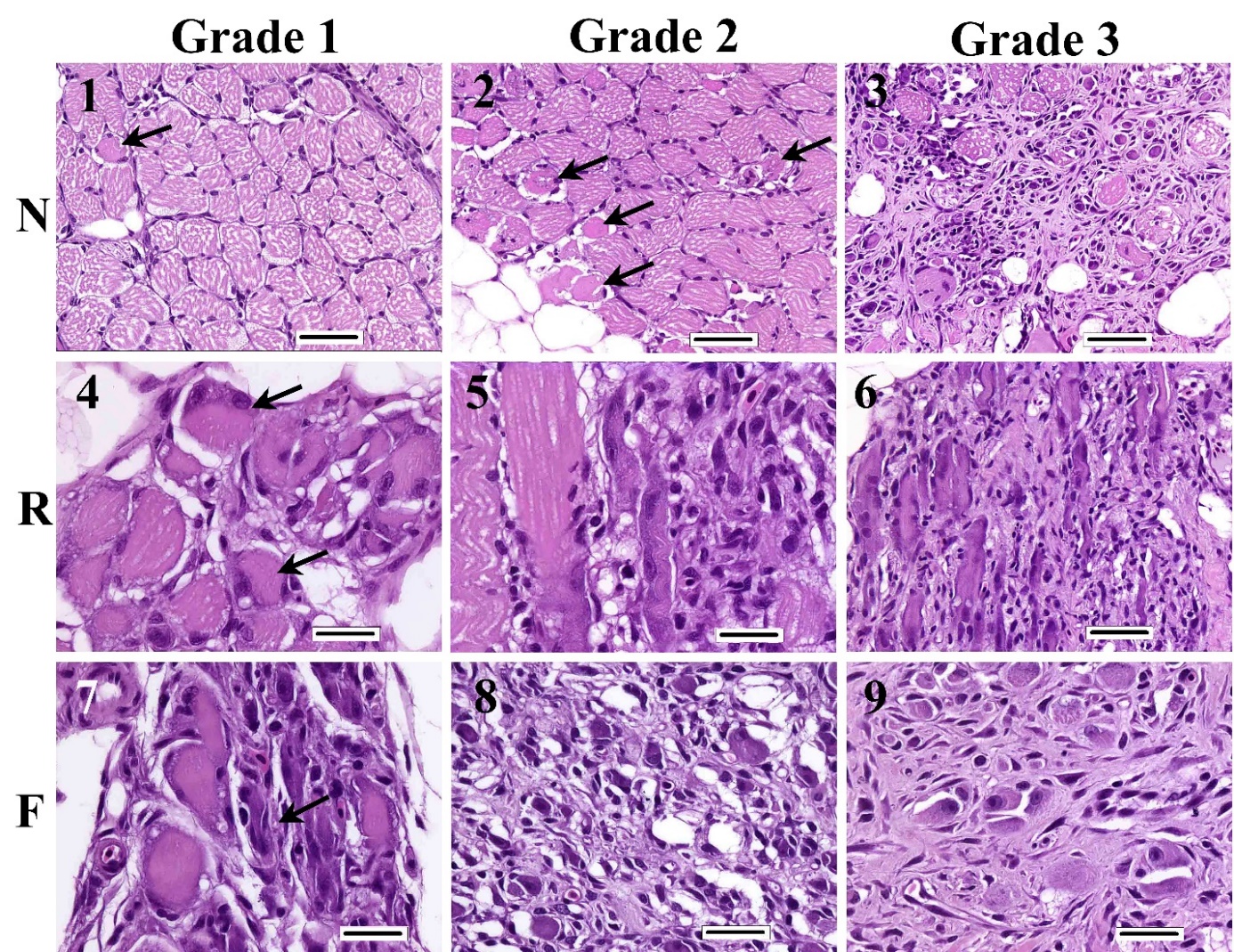
**

**Figure S3.** Severity grading examples of red muscle findings. 1) Arrow indicates a single necrotic myocyte. 2) Arrows indicate several necrotic myocytes in a high magnification field. 3) Essentially all remaining myocytes in this image are necrotic. 4) A couple of myofibers (arrows) have plump basophilic satellite cell nuclei that are beginning to internalize. 5) Myocyte regeneration is widespread and characterized by elongated multinucleated myofibers, but a couple of unaffected fibers (left) in this image are still evident. 6) Essentially all myofibers in this image are undergoing regeneration. 7) A few small strands of collagenous fibers (arrow) and proliferating fibroblasts can be seen between regenerating myofibers. 8) In patchy areas, regenerating myofibers are diffusely separated by loose fibrous connective tissue. 9) Much of the skeletal muscle is replaced by dense fibrous connective tissue. Severity grading criteria for white muscle were similar to those for red muscle, and are therefore not included. N = necrosis, R = regeneration, F = fibrosis. Bar sizes: images 4-5 and 7-9, bar = 25 mm; images 1-3 and 6, bar = 50 mm.
