# Supplementary material for: Effect of pancreas disease vaccines on infection levels and virus transmission in Atlantic salmon (Salmo salar) challenged with salmonid alphavirus, genotype 2
Source: Front Immunol. 2024 Mar 7;15:1342816. doi: 10.3389/fimmu.2024.1342816 (PMC10955579; doi:10.3389/fimmu.2024.1342816)
Supplement: Supplementary file 1 [file DataSheet_1.zip › Supplementary Figure 4.DOCX]

**
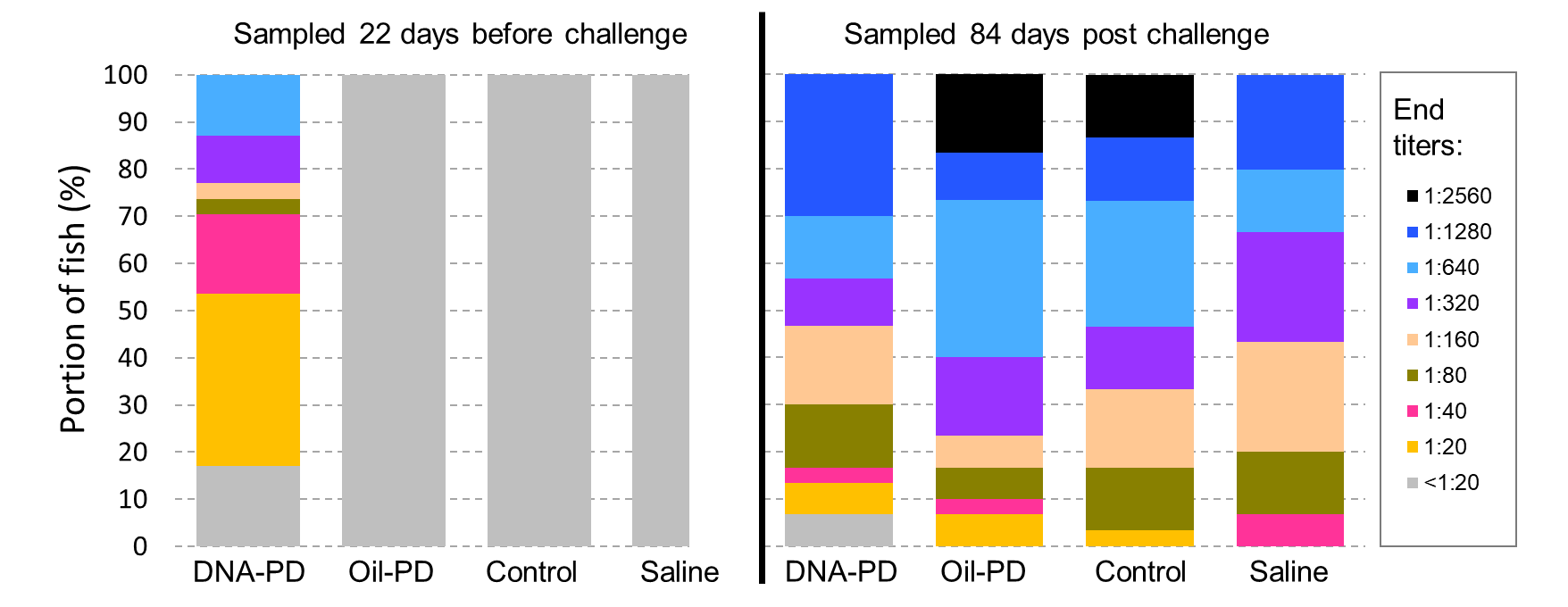
**

**Figure S4.** Neutralization end titres from plasma sampled 22 days before and 84 days post challenge (dpc). n=30 per group per sampling timepoint.
