# Supplementary material for: Effect of pancreas disease vaccines on infection levels and virus transmission in Atlantic salmon (Salmo salar) challenged with salmonid alphavirus, genotype 2
Source: Front Immunol. 2024 Mar 7;15:1342816. doi: 10.3389/fimmu.2024.1342816 (PMC10955579; doi:10.3389/fimmu.2024.1342816)
Supplement: Supplementary file 1 [file DataSheet_1.zip › Supplementary Figure 6.DOCX]

**
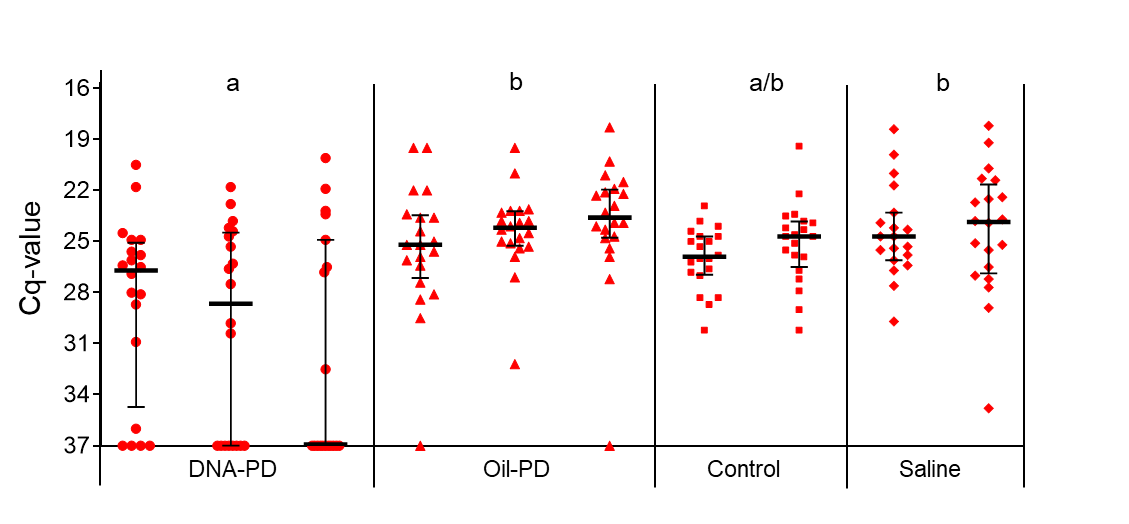
**

**Figure S6.** RT-qPCR SAV2 results showing Cq-values from hearts of the pre-challenged fish sampled 47 dpc (19-20 fish/tank) including medians with interquartile ranges (same timepoint as 26 dpe of the cohabitant fish groups). For the DNA-PD and Oil-PD groups, the dots represent fish that resided with naïve- (left, TS1), with vaccinated- (middle, TS2) or without any cohabitant fish (right). For the Control and Saline groups, the dots represent fish that resided with naïve- (left) or without any cohabitant fish (right). Different letters (a, b) denote significant differences between the groups with the cohabitants adjusted for (Quantile regression analysis p<0.001). The vertical axis value of 37 denotes the cut-off cycle for the qPCR.
