# Supplementary material for: Effect of pancreas disease vaccines on infection levels and virus transmission in Atlantic salmon (Salmo salar) challenged with salmonid alphavirus, genotype 2
Source: Front Immunol. 2024 Mar 7;15:1342816. doi: 10.3389/fimmu.2024.1342816 (PMC10955579; doi:10.3389/fimmu.2024.1342816)
Supplement: Supplementary file 1 [file DataSheet_1.zip › Supplementary Figure 7.DOCX]

**
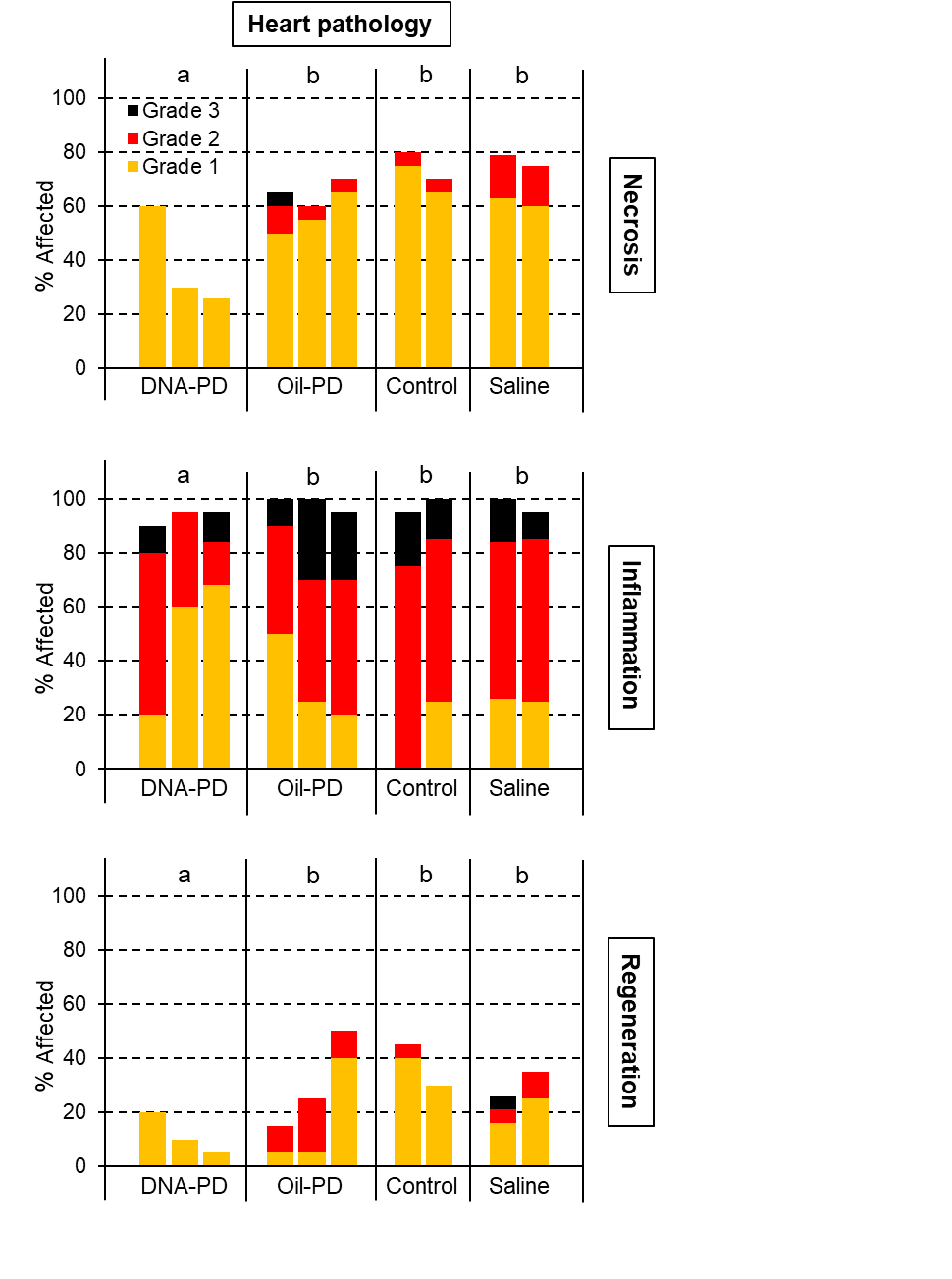
**

**Figure S7.** The prevalence and severity of necrosis, inflammation and regeneration in the hearts of the pre-challenged fish used in the transmission studies sampled at 47 dpc (n= 19-20/group and tank). For the DNA-PD and Oil-PD groups, the bars represent fish that resided with naïve- (on left, TS1), with vaccinated- (in middle, TS2) or without any cohabitant fish (right). For the Control and Saline groups, the bars represent fish that resided with naïve (on left, TS1) or without any cohabitant fish (right). Different letters (a and b) denote significant differences when adjusted for the cohabitation groups (Ordinal logistic regression p<0.02). Note that the total height of each bar represents the overall prevalence of each finding.
