# Supplementary material for: Effect of pancreas disease vaccines on infection levels and virus transmission in Atlantic salmon (Salmo salar) challenged with salmonid alphavirus, genotype 2
Source: Front Immunol. 2024 Mar 7;15:1342816. doi: 10.3389/fimmu.2024.1342816 (PMC10955579; doi:10.3389/fimmu.2024.1342816)
Supplement: Supplementary file 1 [file DataSheet_1.zip › Supplementary Figure 8.DOCX]

**
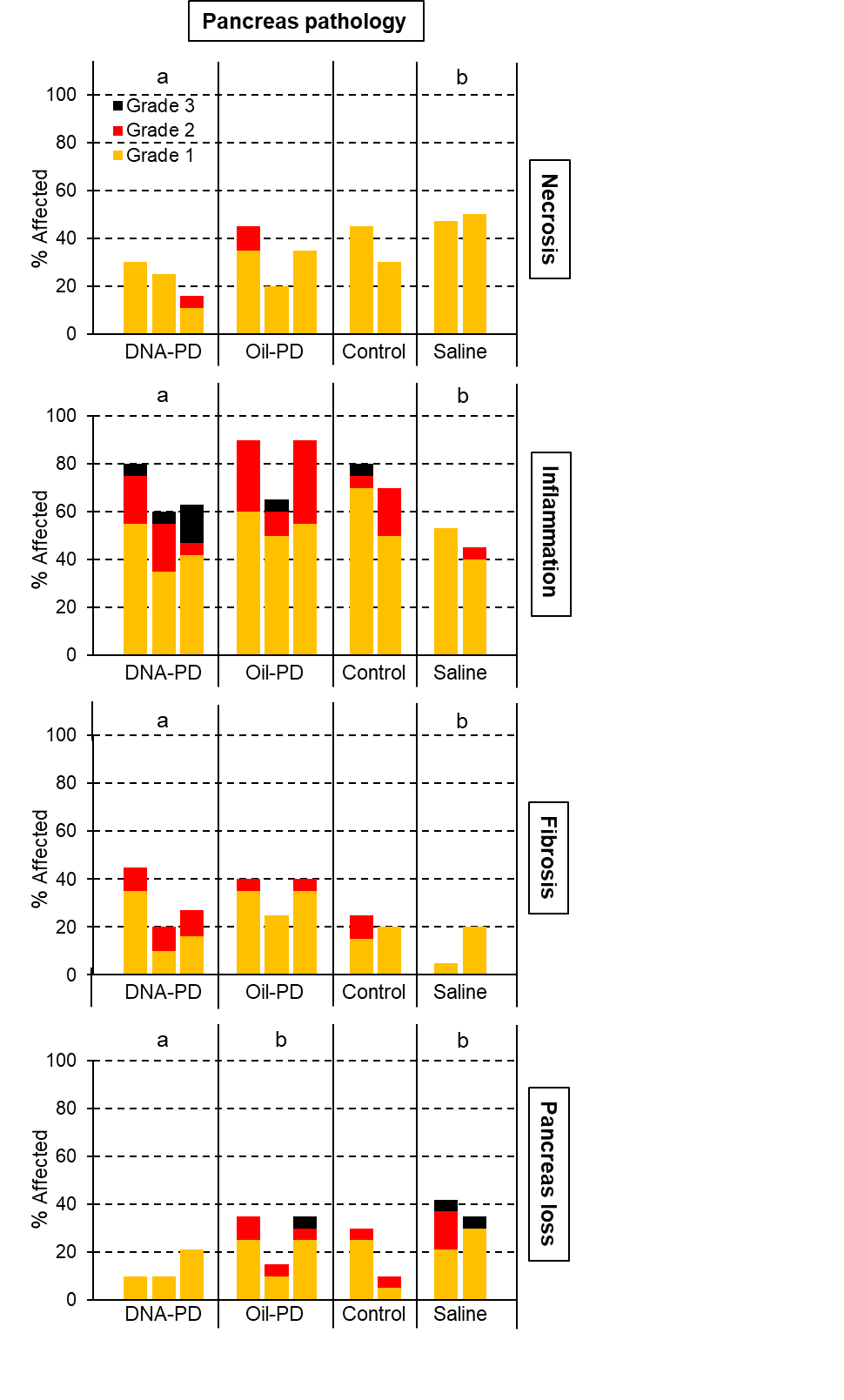
**

**Figure S8.** The prevalence and severity of necrosis, inflammation, fibrosis and tissue loss in the pancreas of the pre-challenged fish used in the transmission studies sampled at 47 dpc (n= 19-20/group and tank). For the DNA-PD and Oil-PD groups, the bars represent fish that resided with naïve- (on left, TS1), with vaccinated- (in middle, TS2) or without any cohabitant fish (right). For the Control and Saline groups, the bars represent fish that resided with naïve- (on left, TS1) or without any cohabitant fish (right). Different letters (a and b) denote significant differences when adjusted for the cohabitation groups (Ordinal logistic regression p<0.05). Note that the total height of each bar represents the overall prevalence of each finding.
