# Supplementary figures and images for: Effect of pancreas disease vaccines on infection levels and virus transmission in Atlantic salmon (Salmo salar) challenged with salmonid alphavirus, genotype 2
Source: Front Immunol. 2024 Mar 7;15:1342816. doi: 10.3389/fimmu.2024.1342816 (PMC10955579; doi:10.3389/fimmu.2024.1342816)

**
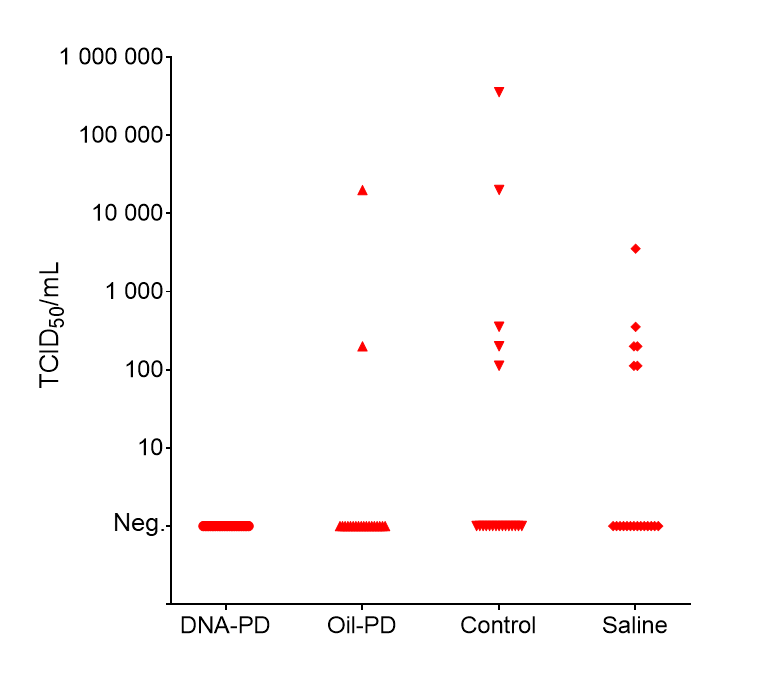
**

**Figure S5.** Viremia levels in the Efficacy study at 19 dpc (n= 20/group).

Supplement: Supplementary file 1 [file DataSheet_1.zip › Supplementary Figure 5.DOCX]
